# *In silico* and *in vitro* drug screening identifies new therapeutic approaches for Ewing sarcoma

**DOI:** 10.18632/oncotarget.13385

**Published:** 2016-11-16

**Authors:** Ziyan Y. Pessetto, Bin Chen, Hani Alturkmani, Stephen Hyter, Colleen A. Flynn, Michael Baltezor, Yan Ma, Howard G. Rosenthal, Kathleen A. Neville, Scott J. Weir, Atul J. Butte, Andrew K. Godwin

**Affiliations:** ^1^ Departments of Pathology and Laboratory Medicine, Toxicology and Therapeutics, University of Kansas Medical Center, Kansas City, KS, USA; ^2^ Institute for Computational Health Sciences, University of California, San Francisco, San Francisco, CA, USA; ^3^ Lead Development Optimization Shared Resource, University of Kansas Cancer Center, Biotechnology Innovation and Optimization Center, Lawrence, KS, USA; ^4^ University of Kansas Cancer Center, University of Kansas Medical Center, Kansas City, KS, USA; ^5^ Department of Pediatrics, Arkansas Children's Hospital, Little Rock, AR, USA; ^6^ Department of Pharmacology, University of Kansas Medical Center, Kansas City, KS, USA; ^7^ Institute for Advancing Medical Innovation, University of Kansas Medical Center, Kansas City, KS, USA

**Keywords:** Ewing sarcoma, drug repurposing, auranofin, ganetespib, high-throughput screening

## Abstract

The long-term overall survival of Ewing sarcoma (EWS) patients remains poor; less than 30% of patients with metastatic or recurrent disease survive despite aggressive combinations of chemotherapy, radiation and surgery. To identify new therapeutic options, we employed a multi-pronged approach using *in silico* predictions of drug activity via an integrated bioinformatics approach in parallel with an *in vitro* screen of FDA-approved drugs. Twenty-seven drugs and forty-six drugs were identified, respectively, to have anti-proliferative effects for EWS, including several classes of drugs in both screening approaches. Among these drugs, 30 were extensively validated as mono-therapeutic agents and 9 in 14 various combinations *in vitro*. Two drugs, auranofin, a thioredoxin reductase inhibitor, and ganetespib, an HSP90 inhibitor, were predicted to have anti-cancer activities *in silico* and were confirmed active across a panel of genetically diverse EWS cells. When given in combination, the survival rate *in vivo* was superior compared to auranofin or ganetespib alone. Importantly, extensive formulations, dose tolerance, and pharmacokinetics studies demonstrated that auranofin requires alternative delivery routes to achieve therapeutically effective levels of the gold compound. These combined screening approaches provide a rapid means to identify new treatment options for patients with a rare and often-fatal disease.

## INTRODUCTION

Ewing sarcoma (EWS) is a rare, aggressive malignancy of neuroectodermal origin that develops in bones and, less often, in soft tissues [1]. EWS is the second most common bone malignancy after osteosarcoma [2], and it frequently develops in pediatric and young adult age groups [3]. The incidence of EWS is about 3/1,000,000 cases per year, with a clear predominance in Caucasians. The American Cancer Society estimates that 225 children and adolescents are diagnosed with EWS in North America each year. The most significant prognostic factor for patients with EWS is the presence or absence of overt metastatic disease. Metastatic disease is most commonly located in the lungs (30%), bone and/or bone marrow (30%), and lung metastasis combined with bone and/or bone marrow metastasis (20%) [4]. Other clinical prognostic factors have been found, such as tumor location, tumor size, patient age, and pattern of metastasis [5]. About 25% of patients show distant metastasis at the time of diagnosis [6]. More than 90% of the EWS tumors harbor an (11;22) (q24;q12) translocation that encodes for a EWS/FLI1 fusion protein [7], which functions as a potent oncoprotein with an abnormal transcription factor behavior that leads to aberrant expression of numerous genes and contributes to tumorigenicity [8, 9].

The current therapies for patients with metastatic EWS are limiting. The introduction of the multimodal approach in the management of EWS resulted in a significant increase in survival for EWS patients. Adding chemotherapy to the combination of surgery and radiation is essential in treating EWS due to the presence of residual tumor after resection and micro-metastasis even in localized disease [10]. The front-line chemotherapy for EWS is alternating vincristine/doxorubicin/cyclophosphamide (VDC) and ifosfamide/etoposide (IE) [11]. Patients with localized tumors currently have a 75% survival rate compared to 10% before the introduction of chemotherapy. Unfortunately, patients with metastatic disease (about 25% of patients at the time of diagnosis) still do poorly with a 20% 5-year-survival rate [7, 10, 12]. A high percentage of patients develop metastasis and resistance to the current treatment regimens [10], which warrants further exploration of possible therapeutic targets.

## RESULTS

### *In silico* prediction

**Figure 1:**
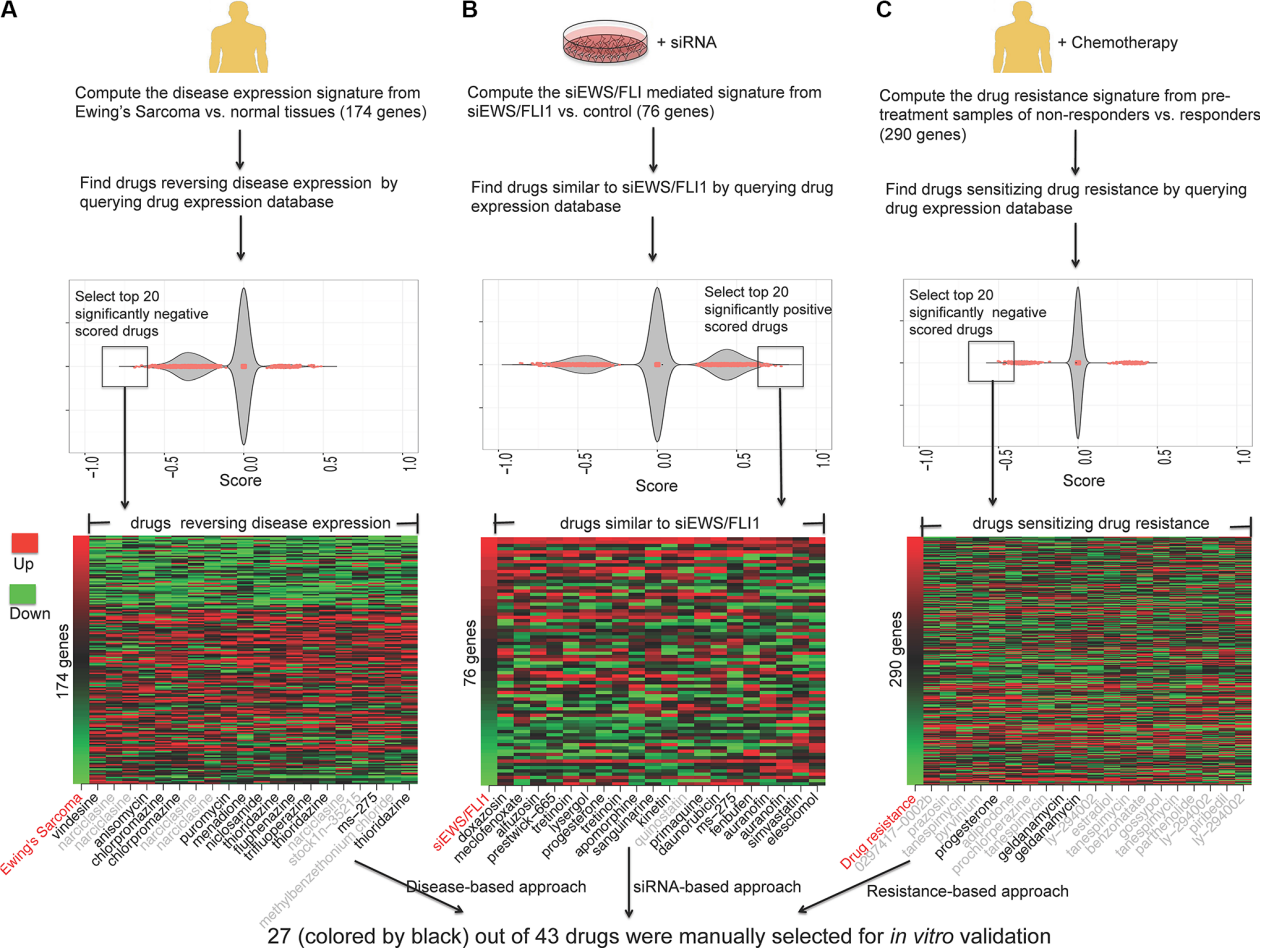
*In silico* prediction of drugs for EWS using three approaches (**A**) Identify drugs that are likely to reverse disease gene expression (disease-based approach). A disease gene expression signature was created from the results of two meta-analysis studies [75, 76], where disease tissue samples and normal tissue samples were compared. (**B)** Identify drugs that mediate gene expression similar to siRNA *EWS/FLI1* (siEWS/FLI1) (siRNA-based approach). The siEWS/FLI signature was taken from the previous study [77]. (**C**) Identify drugs that are likely to sensitize drug resistance expression (resistance-based approach). A drug resistance expression signature was computed by comparing the pre-treatment samples of patients who responded to chemotherapy versus those who did not respond to chemotherapy [78]. Drug gene expression databases were built from the CMap 2.0 and part of the LINCS. In the plot, each dot represents the score of one drug instance. One drug may have multiple instances due to multiple experiments. False discovery rate (FDR) value < 0.05 was used to select drug hits and only the top 20 drug hits for each approach were selected. In the heat map, the first column represents the disease gene expression ranked by fold change and the remaining columns represent the gene expression of drug hits. Red shows up-regulated genes and green shows down-regulated genes. All the drug hits from the three approaches were merged and manually evaluated. The drugs colored by black were selected for further experimental validation.

The systems approach to repurposing drugs has recently gained momentum in pursuit of new treatments for various diseases [13, 14]. The basic concept is to use integrated bioinformatics to identify drugs that can reverse the gene expression of a given disease. We extended the concept and proposed three computational approaches to predict drugs for EWS from a drug library consisting of 1,335 drugs (Figure 1). First, the top 20 negatively scored drug hits that are predicted to reverse the EWS disease gene expression were selected (including 13 distinct drugs). Second, the top 20 positively scored drug hits (including 18 distinct drugs) that share a gene expression profile similar to the pattern obtained by the silencing of *EWS/FLI1* via RNAi approaches (siEWS/FLI1) were identified. Third, the top 20 drug hits (including 14 distinct drugs) that are predicted to reverse the derived drug resistance expression signature were selected. Some drug hits were repeated within each approach or shared among multiple approaches. For example, the HSP90 inhibitors, geldanamycin and tanespimycin (17-(allylamino)-17-demethoxygeldanamycin, 17-AAG), appeared 2 and 4 times, respectively, in the list of the resistance-based approach. MS-275, an HDAC inhibitor, appeared in both disease-based and siRNA-based approach. In total, 43 distinct drugs were predicted using the three computational approaches. Twenty-seven of these drugs were then manually selected for extensive *in vitro* validation (Figure 1).

### *In vitro* screening

We screened, in parallel to the three computational approaches, an FDA-approved drug library containing 2,316 drugs (1,536 unique chemical entities). A panel of sarcoma cell lines including 3 EWS lines, each with a confirmed *EWS* gene rearrangements [15] and two non-tumorigenic and one benign osteoid osteoma control cell lines (Hs 822.T., Hs 863.T., and Hs 919.T.), respectively were used (Tables 1 and 2). Hs 882.T. and Hs 863.T. were previously reported to be of Ewing sarcoma origin [16]; however, FISH analysis and molecular characterization by our group failed to identify an *EWS* gene rearrangement (unpublished data). The drug screening study protocol employed is summarized in Supplementary Table S1. In total, 78 drugs showed activity in at least one or more EWS cell lines; however, 29 of the compounds were cytotoxic to the non-tumorigenic control cells while 3 drugs (vincristine, doxorubicin and etoposide) are currently prescribed for the treatment of EWS and were eliminated from further consideration. Overall, forty-five (45) drugs were identified in the primary screen, including auranofin (Ridaura^®^) (Table 2) and were nominated for further validation.

**Table 1:** EWS fusion status for EWS and non-tumorigenic cell lines

Cell Line	EWS Fusion Status
A673	EWS/FLI1 Type I
TC-71	EWS/FLI1 Type I
SK-ES-1	EWS/FLI1 Type II
RD-ES	EWS/FLI1 Type II
CHLA-258	EWS/FLI1 Type III
COG-E-352	EWS/ERG fusion
Hs 822.T	No fusion detected
Hs 863.T	No fusion detected
Hs 919.T	No fusion detected

**Table 2:** *In vitro* drug screen hits

Classes	Drug	% Inhibition			
		Hs 919.T.	RD-ES	SK-ES-1	A673
Camptothecins	Topotecan HCl	< 50	97	96	97
Camptothecins	Irinotecan Hydrochloride	< 50	99	98	83
Alkylating agents	Melphalan	< 50	95	91	< 50
Taxanes	Paclitaxel/Taxol	< 50	97	96	96
Gold compound	Auranofin	< 50	100	99	98
Estrogen	Estradiol	< 50	< 50	< 50	65
17-alpha-alkylated anabolic steroid	Methyltestosterone	< 50	73	< 50	< 50
Acetanilides; Steroids and Steroid Derivatives	Vorinostat	< 50	61	< 50	< 50
Nitrofurans	Nitrofural/Nitrofurazone	< 50	52	72	52
Diphenhydramines	Clemastine	< 50	< 50	< 50	60
organo-selenium	Ebselen	< 50	95	96	93
Pterins; Keto-Acids	Methotrexate/Amethopterin (R,S)	< 50	82	< 50	89
Amino Acids	L-Glutamic acid, N-[4-[[(2,4-diamino-6-pteridinyl)methyl]methylamino]benzoyl]	< 50	77	57	85
Carbohydrates	Cytosine β-D-arabinofuranoside/Cytarabine	< 50	93	98	84
Macrocyclic lactone	Rapamycin	< 50	62	51	67
Salicylates and Derivatives; Benzene and Derivatives; Benzyl Esters; Benzylacetates; Methoxyphenols	Mycophenolic Acid	< 50	52	< 50	< 50
Benzylisoquinolines	Cycloheximide	< 50	74	81	77
	(+)-Tubocurarine chloride	< 50	51	< 50	< 50
	Atractyloside potassium salt	< 50	98	99	99
Thienopyridines	Ticlopidine	< 50	< 50	< 50	66
Inhibitor of electron transfer at complex III	Antimycin A	< 50	69	< 50	< 50
	Pyrvinium pamoate	< 50	98	96	84
Pyridine derivatives/analogs	Isoniazid	< 50	< 50	< 50	56
	Trifluridine	< 50	71	< 50	< 50
	Floxuridine	< 50	92	56	68
Purines and purine derivatives	Azathioprine	< 50	< 50	52	< 50
	Mercaptopurine	< 50	< 50	57	< 50
	Thioguanosine	< 50	< 50	64	73
	Clofarabine	< 50	99	98	93
	Cladribine	< 50	99	89	87
	Azaguanine-8	< 50	< 50	< 50	83
	Cyclocytidine	< 50	83	98	86
Benzimidazoles	Albendazole	< 50	93	93	89
	Fenbendazole	< 50	86	78	75
	Mebendazole	< 50	95	96	94
	Flubendazole	< 50	86	86	77
	Oxibendazole	< 50	80	84	64
Enzyme inhibitor	Oseltamivir	< 50	95	98	95
	Disulfiram	< 50	96	95	99
	Raltitrexed	< 50	80	51	59
	Novobiocin sodium	< 50	51	< 50	60
	Amorolfine	< 50	< 50	< 50	56
	Camptothecine (S,+)	< 50	97	96	99
	AM 404	< 50	< 50	< 50	65
	Etoposide	< 50	84	84	< 50

### Post screen analysis and secondary screen

**Figure 2:**
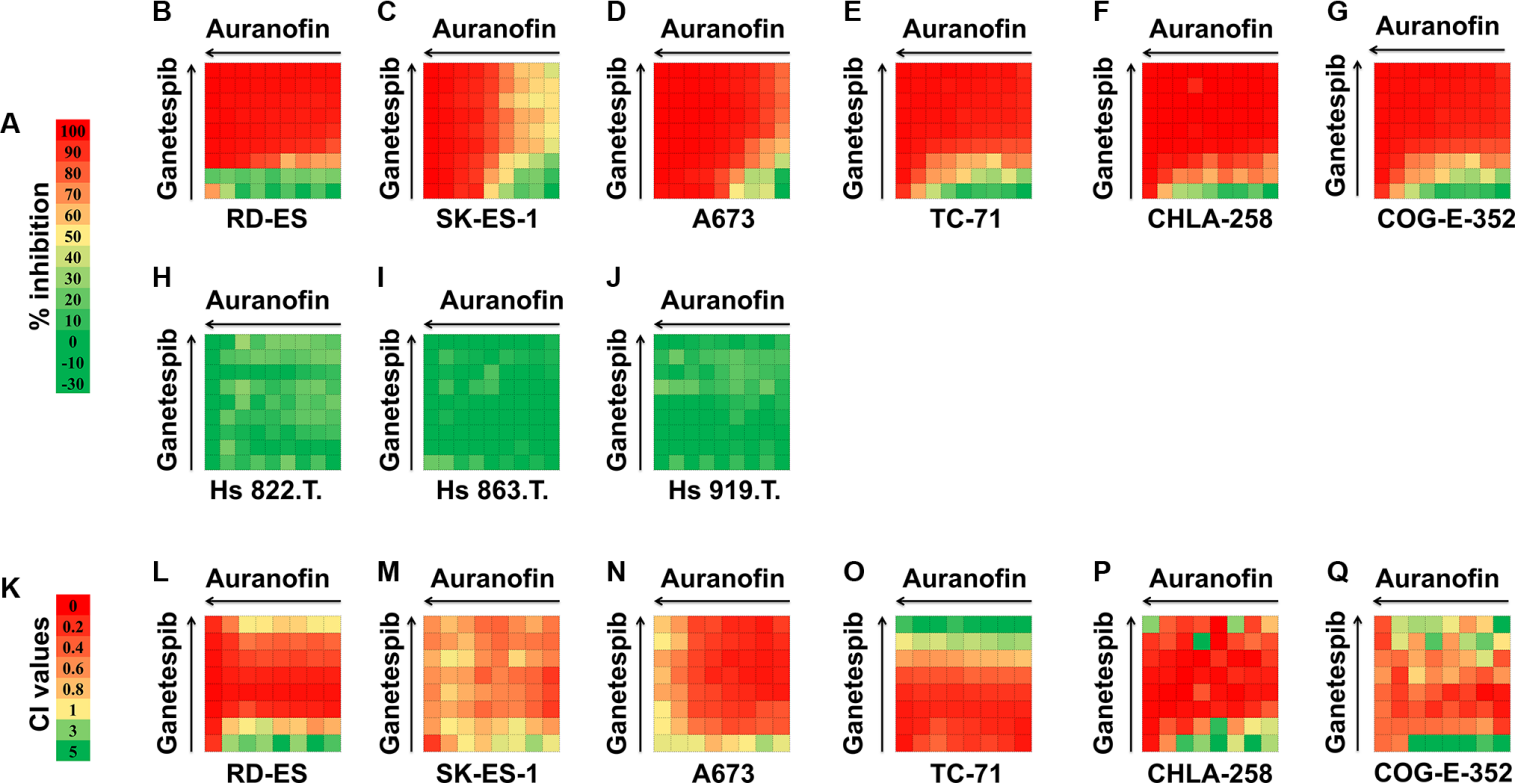
Combination analysis for auranofin and ganetespib in six EWS cell lines and three control (Hs 822.T., Hs 863.T., and Hs 919.T.) cell lines Inhibitory concentration values for individual auranofin or ganetespib as well as drug combinations are shown. (**A**) Color scale for drug inhibition values. (**B**–**J**) Synergy between auranofin and ganetespib was tested by CellTiter-Glo assay at 64 different drug combinations for each cell line. (**K**) Color scale for Combination Index (CI) values. (**L**–**Q**) CI values for auranofin and ganetespib combination treatment for six EWS cell lines. CI value of > 1 indicates antagonism effects; CI = 1 indicates additive effects; CI value of < 0.9 indicates synergy effects; and CI value of < 0.5 indicates strong synergy effects. (Negative inhibition values cannot be used to calculate CI values).

Next, the predicted and library screened drugs were extensively validated. The *in vitro* activities of each drug hit were evaluated in an expanded panel of EWS cell lines (either the *EWS/FLI1* or the *EWS/ERG* fusion) (Table 1) and confirmed employing 12-point dose-response curves ranging from 50 μmol/L to 1 nmol/L, with triplicate sampling (Table 3). As mentioned above, one of the most notable drug hits discovered through both the *in silico* and *in vitro* drug screening approaches was auranofin. Based on this finding, we subsequently screened auranofin against six other marketed or investigational anticancer drug hits (carfilzomib, elesclomol, bortezomib, geldanamycin, AUY922, and ganetespib, Supplementary Figures S1–S5 and Figure 2). These drugs were chosen based on their known mechanisms and safety profiles. We also tested the synergy between these drug hits (Supplementary Figures S6–S11). Several classes of agents were found to be highly synergistic in combination with auranofin, including the next generation HSP90 inhibitors ganetespib (STA-9090; Synta Pharmaceuticals) and AUY922 (Novartis). These independent results peaked our interest since geldanamycin and its derivative 17-AAG were repeatedly identified in our integrated bioinformatics studies. Since the development of geldanamycin and 17-AAG was discontinued due to clinical safety issues observed in clinical trials [17], we sought to investigate a lead HSP90 inhibitor, ganetespib (Synta Pharmaceuticals), which has a better risk-benefit profile in clinical trials. We evaluated the anti-proliferation effect in combination treatments using the EWS and control cell lines. As shown in Figure 2, the combined treatment of auranofin and ganetespib inhibited cell growth and was highly synergistic across all of the EWS lines as opposed to the non-tumorigenic cell lines (red shows strong *in vitro* activity while green lack thereof). The above-mentioned screening results were deemed sufficient to warrant *in vivo* preclinical proof of principle studies.

**Table 3:** *In vitro* validation of drug hits

Drugs	IC_50_ (μmol/L)								
	A673	RD-ES	SK-ES-1	TC-71	CHLA-258	COG-E-352	Hs 822.T.	Hs 863.T.	Hs 919.T.
**Vindesine^1^**	< 0.003	< 0.003	< 0.003	NR, < 0.003	NR, < 0.003	< 0.003	0.25 ± 0.01	0.28 ± 0.01	4.58 ± 0.45
**Elesclomol^2^**	0.006 ± 0.006	0.05 ± 0.06	0.01 ± 0.001	< 0.003	NR, < 0.003	0.007 ± 0.002	> 20	> 20	> 20
**Niclosamide^1^**	7.02 ± 1.23	8.54 ± 1.55	12.5 ± 2.36	0.79 ± 0.47	0.70 ± 0.14	0.42 ± 0.07	15.24 ± 3.51	10.45 ± 2.66	10.56 ± 2.84
**Sanguinarine^2^**	0.75 ± 0.14	1.50 ± 0.12	1.87 ± 0.51	0.20 ± 0.08	0.95 ± 0.31	1.05 ± 0.18	1.55 ± 0.26	1.25 ± 0.14	1.44 ± 0.32
**Doxazosin^2^**	5.60 ± 1.52	> 25	20.44 ± 5.87	22.5 ± 9.35	16.14 ± 6.78	17.12 ± 6.58	15.65 ± 2.12	14.58 ±3.02	18.48 ± 3.27
**Fluphenzaine^1^**	10.52 ± 2.89	14.26 ± 3.21	16.27 ± 3.66	17.00 ± 6.68	18.98 ± 6.79	16.14 ± 7.51	22.65 ± 7.23	20.54 ± 2.88	15.47 ± 3.68
**Menadione^1^**	6.80 ± 1.35	10.55 ± 3.20	5.56 ± 1.24	2.94 ± 3.48	3.64 ± 2.08	5.62 ± 1.31	12.20 ± 2.12	14.25 ± 3.20	15.82 ± 2.35
**Puromycin^1^**	0.25 ± 0.01	0.35 ± 0.03	0.35 ± 0.10	0.08 ± 0.05	0.06 ± 0.02	0.05 ± 0.01	0.55 ± 0.13	1.02 ± 0.26	0.89 ± 0.03
**Alfuzosin^2^**	> 50	> 50	> 50	> 50	> 50	> 50	> 50	> 50	> 50
**Daunorubicin^2^**	0.05 ± 0.02	0.05 ± 0.03	0.04 ± 0.03	0.0046 ± 0.0004	< 0.003	< 0.003	0.15 ± 0.12	0.18 ± 0.08	0.42 ± 0.15
**MS275^1,2^**	2.5 ± 1.02	2.0 ± 0.85	2.4 ± 1.01	1.53 ± 0.49	0.15 ± 0.03	1.47 ± 0.54	>25	> 25	> 50
**Simvastatin^2^**	0.58 ± 0.05	3.25 ± 0.14	3.86 ± 0.89	4.33 ± 3.86	0.25 ± 0.13	10.47 ± 1.70	2.08 ± 0.84	4.10 ± 0.56	2.88 ± 0.58
**Auranofin^2^**	0.33 ± 0.22	2.45 ± 0.52	0.08 ± 0.32	0.26 ± 0.14	0.38 ± 0.19	0.82 ± 0.19	1.45 ± 0.09	3.25 ± 0.12	5.38 ± 0.89
**Fenbufen^2^**	> 50	> 50	> 50	> 50	> 50	> 50	> 50	> 50	> 50
**Lysergol^2^**	> 50	> 50	> 50	> 50	> 50	> 50	> 50	> 50	> 50
**Thioridazine^1^**	10.05 ± 1.25	15.02 ± 1.45	15.86 ± 2.47	15.90 ± 1.38	16.06 ± 5.52	10.6 ± 4.37	8.50 ± 1.58	9.24 ± 2.01	8.84 ± 1.29
**Trifluoperazine^1^**	15.60 ± 3.55	18.21 ± 2.54	16.68 ± 3.14	16.38 ± 3.46	13.63 ± 4.80	12.10 ± 1.58	10.55 ± 2.45	9.84 ± 2.28	16.82 ± 4.20
**(R)-Apomorphine^2^**	8.52 ± 2.14	7.85 ± 1.69	10.55 ± 3.20	17.30 ± 2.21	14.67 ± 6.86	4.01 ± 1.55	14.32 ± 1.47	15.22 ± 2.19	16.68 ± 3.02
**Meclofenoxate^2^**	> 50	> 50	> 50	> 50	> 50	> 50	> 50	> 50	> 50
**Chlorpromazine^1^**	10.57 ± 2.15	> 25	> 25	17.61 ± 8.63	15.27 ± 4.55	16.14 ± 2.29	16.80 ± 3.22	18.40 ± 3.07	15.49 ± 2.09
**Tretinoin^2^**	> 50	> 50	> 50	> 50	4.95 ± 3.82	35.54 ± 6.28	> 50	> 50	> 50
**Kinetin^2^**	> 50	> 50	> 50	> 50	> 50	> 50	> 50	> 50	> 50
**Evoxine^2^(prestwick−665)**	> 50	> 50	> 50	> 50	> 50	> 50	> 50	> 50	> 50
**Primaquine^2^**	32.52 ± 6.58	> 25	> 25	14.28 ± 3.71	7.14 ± 2.21	13.33 ± 4.09	> 25	> 25	> 50
**Progesterone^2,3^**	> 50	> 50	> 50	> 50	11.5 ± 5.31	24.88 ± 5.85	> 50	> 50	> 50
**Anisomycin^1^**	0.12 ± 0.01	0.15 ± 0.02	0.20 ± 0.01	0.04 ± 0.01	0.08 ± 0.01	0.02 ± 0.01	0.15 ± 0.05	0.20 ± 0.01	0.15 ± 0.02
**Bortezomib^4^**	0.01 ± 0.003	0.01 ± 0.001	0.02 ± 0.002	0.005 ± 0.0081	0.007 ± 0.03	0.005 ± 0.04	0.45 ± 0.08	0.18 ± 0.03	0.17 ± 0.003
**Geldanamycin^3^**	1.93 ± 0.05	0.05 ± 0.003	0.03 ± 0.002	< 0.003	< 0.003	< 0.003	> 20	> 20	> 20
**Genatespib^4^**	0.04 ± 0.01	0.02 ± 0.02	0.01 ± 0.001	< 0.003	< 0.003	< 0.003	> 20	> 20	> 20
**AUY922^4^**	0.02 ± 0.002	0.009 ± 0.001	0.03 ± 0.002	< 0.003	< 0.003	< 0.003	> 20	> 20	> 20

The hits are annotated based on their source:

^1^Disease-based approach;

^2^siRNA-based approach;

^3^Resistance-based approach; or

^4^*In vitro*-based screen and others.

### The expression of EWS-FLI1 influences sensitivity to auranofin

**Figure 3:**
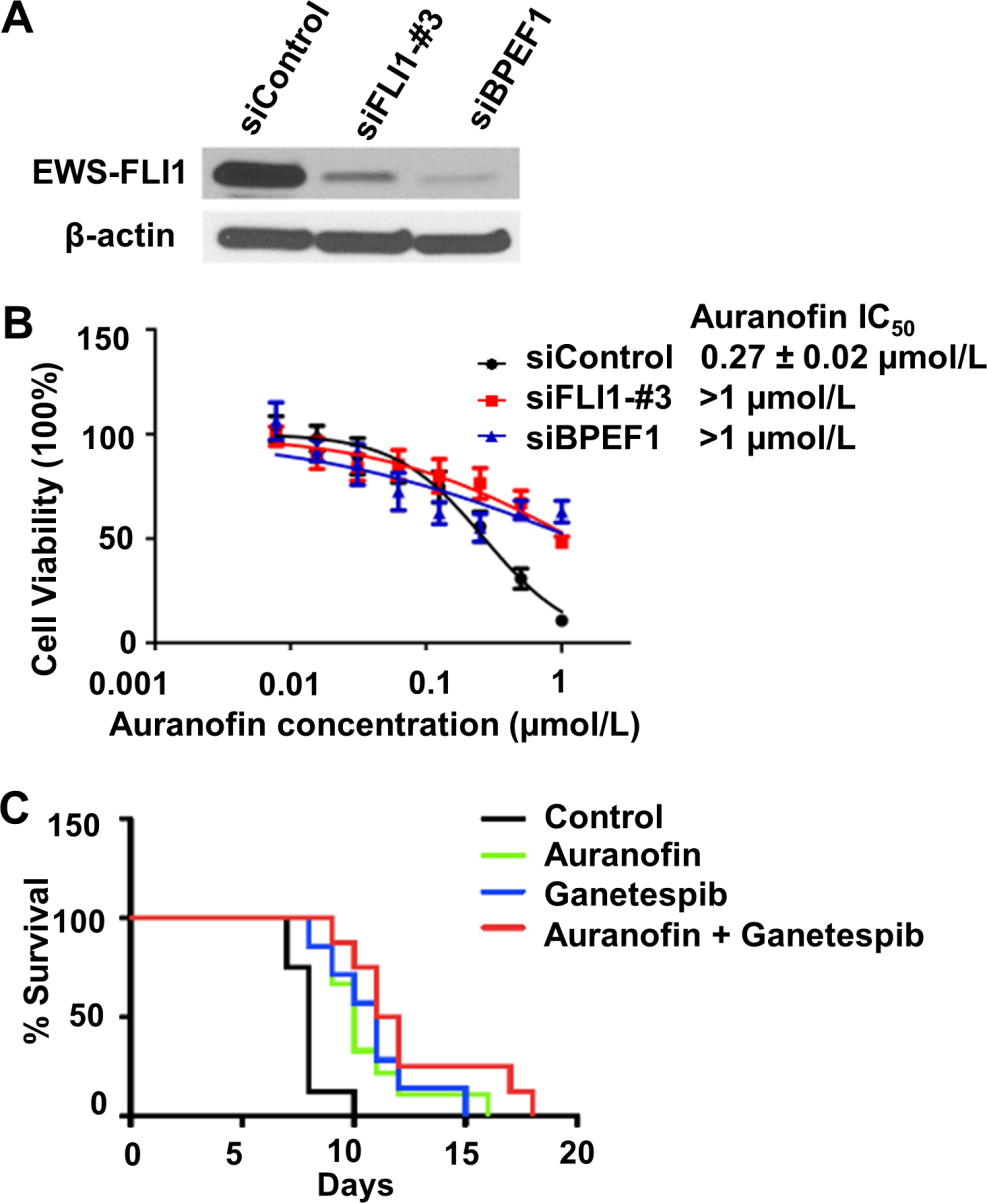
The sensitivity of EWS cells to auranofin is dependent on expression of the *EWS-FLI 1* oncogene (**A**) Representative western blot data shows the significant decrease in the expression of *EWS-FLI1* in A673 cells 48h post-transfection. (**B**) Cell viability was assessed by the CellTiter Blue assay in A673 cells treated by siControl, siFLI1-#3 or siBPEF1 for 48 h then followed by the incubation of auranofin ranging from 7.8 nmol/L to 1 μmol/L for 72h. Data are presented as the mean ± SD of three independent experiments performed in triplicate. (**C**) *In vivo* studies of auranofin, ganetespib and the combination treatment in an EWS xenograft mice model (*n* = 12 per group). The percentage of survival rate in control, auranofin treated, ganetespib treated, and auranofin in combination with ganetespib treated groups.

Because auranofin is predicted to mimic the silencing of *EWS-FLI1* oncogene in the *in silico* analysis and it shows preferential toxicity against Ewing cells harboring the *EWS-FLI1* chimeric gene as compared with the non-tumorigenic cells without the *EWS-FLI1* gene (Figure 1), we then investigated whether Ewing cells are sensitive to auranofin if *EWS-FLI1* is silenced. To knock down the expression of *EWS-FLI1* in A673 Ewing cells, we designed two different siRNAs, corresponding to the sequence within the 3′ downstream half of *EWS-FLI1* mRNA (siFLI1-#3) and the sequence of the breakpoint of *EWS-FLI1* type I mRNA (siBPEF1) respectively. To examine whether the siRNAs could abolish the expression of *EWS-FLI1*, the effects of siRNAs on the protein levels of *EWS-FLI1* were analyzed by western blot (see Supplementary methods). Treatment with siFLI1-#3, or to a greater extent, siBPEF1, significantly decreased the EWS-FLI1 protein expression in A673 cells with type I fusion at 48h after transfection, as compared with the siControl treatment (Figure 3A). In addition, A673 cells became resistant to auranofin after the silencing of *EWS-FLI1* gene, as evidenced by the significant increase in the IC_50_ values of A673 cells treated by siFLI1-#3 or siBPEF1 followed by the incubation of auranofin for 72 h (siFLI1-#3 and siBPEF1 IC50 values are above 1.0 μmol/L), as compared with the counterpart of the siControl group (siControl, IC_50_ = 0.27 ± 0.02 μmol/L) (Figure 3B). These experiments further validate the *in silico* prediction and suggest a need to further assess its potential clinical utility as an anti-cancer agent.

### Defining maximum tolerated dose

Ridaura^®^ (auranofin) is typically delivered orally (capsules containing 3 mg auranofin) and has been repurposed in several clinical trials with limited results (*i.e*., clinicaltrials.gov identifier: NCT01419691, NCT02126527, and NCT02126527). We hypothesized that the oral bioavailability of auranofin (gold) might explain the difference in its anti-cancer activities *in vitro* versus in clinical trials given that the published product monograph for Ridaura^®^ capsules stated that the human oral bioavailability of gold from Ridaura^®^ capsules is only approximately 25% [18, See Supplementary Material Reference 18].. Additionally, the ability to increase the dose of auranofin to achieve higher blood concentrations of gold was limited by dose-limiting gastrointestinal side effects (diarrhea, loose stools, nausea, vomiting and abdominal cramps) [18, 19]. For our studies we dosed BALB/c mice orally at 10 mg/Kg with different formulations of auranofin suspensions prepared in 0.5% hydroxypropyl methyl cellulose to help assure dose uniformity. We compared the ground contents of Ridaura^®^ capsules, the ground pure auranofin drug substance, auranofin suspension containing 0.5% sodium lauryl sulfate (a surfactant), auranofin suspensions with the pH adjusted to pH = 1, 4 and 7, auranofin suspension dosed into fasted versus high fat diet mice and finally a solution of auranofin prepared in 5% dimethyl sulfoxide (DMSO) with a pH = 1. Pharmacokinetic studies were conducted on all of these formulation variations. Blood samples from 3 mice at each time point (0, 1, 2, 4, 8, 12, 24, 48 and 96 hours) and the whole blood was assayed by ICPMS for gold.

We additionally studied dose accumulation of the gold by comparing a formulation of Ridaura^®^ capsule contents as described above and dosing at 10 mg/Kg single dose versus dosing the mice once daily for 5 days. The PK study compared the gold content in whole blood samples as described above for the single dose versus the gold content in whole blood samples from the multidose arm with whole blood collected at the end of each 24 hour dosing period before the next dose (24, 48, 72, 96 and 120 hours) and then whole blood collected every 24 hours for 5 additional days (144, 168, 192, 216 and 240 hours) and assayed for gold content. The results of these formulation and PK studies are shown in Supplementary Table S2.

The formulation studies demonstrated that we were unable to alter the amount of auranofin (gold) absorbed through formulation variations. These findings led us to develop and assess injectable (i.v. and i.p.) formulations of auranofin. By defining maximum tolerated dose (MTD) following i.v. and i.p. administration of solution and suspension formulations of auranofin, our intent was to evaluate auranofin alone and in combination with ganetespib at MTD in mouse xenograft studies using the more convenient i.p. route of administration. Following administration of the solution formulation of auranofin i.v. and i.p., the maximum tolerated dose was estimated at 1 mg/kg and 6 mg/kg, respectively. Following a single i.p. suspension dose of auranofin, the maximum tolerated dose was 16 mg/kg. This dose was reduced to 12 mg/kg and dose tolerance was evaluated after 5 daily doses of auranofin suspension i.p. to determine the effects of the accumulation of gold in the blood due to the slow elimination of gold. Based on the i.p. suspension dose tolerance studies, the auranofin suspension formulation and dose level of 12 mg/kg was selected for evaluation in xenograft mouse studies.

### Auranofin pharmacokinetics

The single-dose pharmacokinetics of auranofin following pre-treatment with i.v. ganetespib were characterized to describe systemic gold exposure near the dose evaluated in EWS mouse xenograft studies. By describing blood gold concentrations *in vivo*, we are able to place the auranofin *in vitro* IC_50_ values described in Table 3, as well as *in vivo* tumor response data in proper context.

As indicated previously, auranofin is a complex of gold triethylphosphine and thioglucose tetraacetate. Gold comprises 29% of auranofin by weight. Auranofin is chemically and metabolically highly unstable in solution. In our studies, auranofin immediately and completely dissociates in plasma and whole blood *ex vivo*, as well as simulated gastric fluid *in vitro* (unpublished results). Following oral administration of ^195^Au auranofin to humans, 25% of administered ^195^Au appears in plasma [19]. Further, approximately 40% of absorbed gold distributes into erythrocytes with 60% found in plasma [19]. As a result, and consistent with the extensive literature describing auranofin pharmacokinetics in animals and humans, we characterized the pharmacokinetics of i.p. auranofin in mice by quantifying blood gold concentrations as summarized in Table 4. Peak blood gold concentrations were 12.43 μg/mL in mice receiving auranofin suspension preceded by i.v. ganetespib. The apparent elimination half-life of gold was 23.7 hours in mice pretreated with ganetespib. The area under the blood gold concentration-time profile, another measure of systemic exposure to gold, was 535 hr*μg/mL in mice pretreated with ganetespib. Following five consecutive once daily i.p. doses of 12 mg/kg auranofin suspension in mice pretreated with ganetespib, the trough (plateau) plasma gold concentration (24 hours following the fifth daily dose) was 23.9 μg/mL.

**Table 4:** Blood gold pharmacokinetic parameters in BALB/c mice following a single i.p. dose of 12 mg/kg auranofin suspension following pre-treatment with ganetespib^a^

Treatment	Auranofin+Ganetespib
Mean Body Weight (kg)	0.02026
Auranofin Dose (mg/kg)^b^	12
Gold Dose (mg/kg)^c^	3.48
C_max_ (μg/mL)	12.43
C_max_ (μmol/L)	6.31
T_max_ (hr)	8.0
AUC_0_∫^∞^ (μg*hr/mL)	536.26
K_el_ (hr^−1^)	0.029
T_½_ (hr)	23.7
Cl/F (mL/hr/kg)	6.49
Trough (μg/mL)^d^	23.87

^a^*N* = 30, *N* = 3 per serial blood collection time point, ten serial blood samples collected over 48 hours.

^b^Auranofin injectable suspension formulation contained 0.67 mg/ml of auranofin and 0.5% HPMC K4M in D5W. Mice were pretreated with 150 mg/kg ganetespib i.v. approximately 24 hours prior to the administration of the single i.p. dose of auranofin.

^c^On a weight basis, auranofin contains 29% gold. Therefore, the i.p. dose of gold administered was 3.48 mg/kg.

^d^Trough sample was collected 24 hours following the fifth once daily i.p. dose.

The mean single dose C_max_ value of 12.43 μg/mL, expressed in molar concentration, equals to 63.10 μmol/L (Table 4). The mean *in vitro* IC_50_ values for auranofin are described in Table 3, for each of the EWS cell lines tested, ranged from 0.08 to 5.38 μmol/L. IC_50_ values for gold in these cell lines would be 29% of the values presented in Table 3. We conclude that systemic drug exposure to gold following 12 mg/kg ip auranofin suspension exceeded the *in vitro* IC_50_ values for auranofin, and for gold, in each of the EWS cell lines studied. In addition, blood gold concentrations achieved in the studies described in this work exceed systemic drug exposure observed in adult and pediatric rheumatoid arthritis patients receiving therapeutic, chronic doses of auranofin [19, 20].

### Auranofin, ganetespib, and the combination treatments improved survival of mice in a xenograft mouse model

To assess the antitumor activity of auranofin (using an alternative delivery approach), ganetespib and the combination treatments *in vivo*, we generated a xenograft nude mouse model by injecting A673 cells intramuscularly proximal to the tibia. Based on the dose tolerance and pharmacokinetic studies described above, we administered auranofin at a tolerable daily i.p. dose that achieved adequate systemic drug exposure. Auranofin was administered with a dose of 12 mg/kg through an i.p. injection once a day for 5 days per week, and ganetespib was administered with a dose of 150 mg/kg through an i.v. injection once weekly. Mice that grew palpable tumors were randomized into four groups: vehicle, auranofin-only, ganetespib-only, and the combination. The weights of the mice were stable among all four arms throughout the study, and no major side effects were observed except for a skin lesion at the site of the i.v. injection at the base of the tail of 2 mice in the ganetespib-only group. The H&E staining of kidney, liver and spleen tissues showed no sign of toxicity. The survival rates were significantly different between the auranofin only, ganetespib only, and the combination versus control groups (*p <* 0.05, confidence interval 90%). The survival rate in the combination group was nearly doubled in this extremely aggressive EWS tumor animal model when compared to the control group (Figure 3C).

## DISCUSSION

Historically, drug development for pediatric cancers has only occurred after agents have shown efficacy in adult tumors. Clofarabine (approved for refractory pediatric acute lymphoblastic leukemia) remains the only anti-neoplastic agent ever approved for pediatric use prior to adult labeling. Moreover, of the 120 new cancer therapeutic agents approved for adults by the year 2003, only 30 have had a pediatric indication. Of these 30 drugs, only 15 acquired subsequent pediatric labeling. These practices have led to limits in the new agents “pipeline” for children and have hindered advancements in the treatment of EWS. In 2003, Hirschfeld *et al*. stated that the best opportunity for survival for patients with EWS was to identify more effective therapies to apply to newly diagnosed patients [21]. Sadly, more than ten years later, this remains true and underscores the need for the development of novel agents.

The Orphan Drug Act defines a rare disease as one that affects less than 200,000 individuals in the United States [22, 23]. Approximately 30 million people in the United States are living with rare diseases, equating to one in every ten Americans. Based on a recent study published by DiMasi *et al*. for the Study of Drug Development, costs to develop a new pharmaceutical agent is estimated to $2870 million (2013 dollars) [24] The Orphan Drug Act provides pharmaceutical companies with incentives to develop and commercialize new treatments for rare diseases. Despite these incentives; however, the development time and costs make it difficult for firms to invest in new therapies for diseases such as EWS. Drug repurposing, exploring new uses for existing (FDA approved) and abandoned drugs, represents an opportunity to advance new treatments to rare disease patients more quickly, and at a lower cost [25–27]. Over the past few years, biotech, academia, government and disease philanthropy organizations generated successful stories using innovative technologies for drug repurposing [28]. The National Center for Advancing Translational Sciences (NCATS) at the NIH also contributed tremendously to the growth in this translational science area. The Learning Collaborative, a partnership between the University of Kansas Cancer Center (KUCC), the Leukemia and Lymphomia Society (LLS) and the NIH's Therapeutics for Rare and Neglected Diseases (TRND) program, has played a leadership role nationally in this effort [27]. We have leveraged our drug repurposing expertise and experiences, along with an innovative *in silico* integrated bioinformatics platform technology (*e.g*., [13, 29, 30]), to not only identify drug repurposing opportunities in EWS, but to advance these agents administered alone and in combination with other anticancer agents through *in vitro* and *in vivo* preclinical proof of principle studies.

A systematic approach using *in silico* prediction combined with a traditional drug screening approach identified numerous drug repurposing opportunities. Molecular profiling data, critical to predicting drug activity *in silico*, is growing rapidly with increasing public access. Researchers are now able to combine genome-wide proteomics, gene expression profiles, and RNA interference data to build computational molecular pathology systems. These molecular pathology models can then be used to determine drug-disease connections using general molecular characteristics [29]. This systematical approach to repositioning drugs has now been applied to many diseases. Examples include topiramate for inflammatory bowel disease [30], trifluoperazine for lung cancer [31], and rapamycin for lymphoid malignancies [32]. By leveraging public datasets, we predicted 27 out of 1,335 drugs for *in vitro* validation. Fifteen out of 27 drugs exhibit IC_50_ values of less than 10 μmol/L in one or more EWS cell lines and 10 drugs exhibit IC_50_ values less than 10 μmol/L in all six EWS cell lines tested. Notably, 6 drugs (auranofin, geldanamycin, vindesine, elesclomol, daunorubicin, and MS-275) have IC_50_ values ten-fold less in EWS cell lines than in the three non-tumorigenic control cell lines. In addition to the high success rate of discovering new drug hits, *in silico* prediction is relatively inexpensive and quick, suggesting its great potential to screen compounds.

In addition to the drugs that are predicted to reverse the disease gene expression, we identified the drugs that share an expression profile similar to the silence of EWS/FLI1, a key causative factor in the pathogenesis of EWS, and the drugs that may overcome clinical drug resistance. Interestingly, the top 20 drug hits among the three approaches have little overlap, although some drugs may be significant in multiple approaches. For example, geldanamycin, a drug predicted by the resistance-based approach, is also significant in both disease-based approach and siRNA-based approach, but it was ranked out of 20. All three approaches led to positive hits, which exhibit IC_50_ values less than 10 μmol/L in EWS cell lines *in vitro*. These results suggested that *in silico* prediction based on different sources is beneficial. Especially, the recent efforts on molecular characterization of EWS and cellular response to drug treatment provide new opportunities to leverage diverse datasets for finding therapeutics for EWS [33–35].

Our *in vitro* drug screen identified 46 hits of drugs. These drugs were of diverse classes from alkylating agents, steroids, estrogens, and enzyme inhibitors, to gold compounds. We prioritized the top 29 hits from *in silico* and *in vitro* screening that were of potential clinical interest and then moved them forward for extensive *in vitro* validation (Table 3).

Among the six most potent drugs from our screens (*i.e*., auranofin, geldanamycin, vindesine, elesclomol, daunorubicin, and MS-275), MS-275 and elesclomol are the other two drugs that we are pursuing. MS-275 (also known as entinostat) is a selective histone deacetylase (HDAC) inhibitor which targets HDAC1 and HDAC3, and it has been demonstrated to be reasonably safe and effective in the treatment of a wide range of cancers, including leukemia, lymphoma, melanoma, non-small-cell lung cancer and breast cancer, both alone or in combination of other therapies [36–42]. Entinostat is currently marketed by Syndax and is in the phase III clinical trial in combination with exemestane for the treatment of patients with hormone receptor-positive advanced breast cancer (NCT02115282). Meanwhile, two active phase II clinical trials where non-small-cell-lung cancer patients will be treated with entinostat before chemotherapy (NCT01935947) and anti-PD1 treatment (NCT01928576) are ongoing, which indicates the feasibility of combination studies of entinostat with other targeted agents as an epigenetic-based therapy in EWS treatment. Elesclomol (STA-4783) is an investigational drug being developed by Synta Pharmaceuticals and GlaxoSmithKline, which exerts potent anticancer activity through the elevation of reactive oxygen species (ROS) levels and is currently in the phase II clinical evaluation as a novel anticancer therapeutic in combination with paclitaxel in the treatment of recurrent or persistent ovarian cancer (NCT00888615). In addition, the Genomics of Drug Sensitivity in Cancer (GDSC) database shows that cancer cells with *EWS-FLI1* mutations are preferentially sensitive to elesclomol as compare with the wide-type counterparts (http://www.cancerrxgene.org/translation/Drug/1031#t_scatter_1031), which is consistent with our *in vitro* drug screens. Further mechanistic studies of entinostat and elesclomol in Ewing sarcoma are underway (Ma and Godwin, personal communications). In addition, the analogs of vindesine and daunorubicin, named vincristine and doxorubicin, respectively, are the first-line chemotherapies for EWS treatment, which further validates our *in silico* and *in vitro* screening approaches.

In these studies we were drawn to Auranofin (Ridaura^®^) since it was a hit in both *in silico* and *in vitro* screening. Auranofin is an oral gold complex, first approved by FDA for the treatment of rheumatoid arthritis in 1985 [43]. However, the mechanism of action of auranofin is not completely understood. In addition to its anti-inflammatory activity, others and we have reported that auranofin is a potent thioredoxin reductase (TrxR) inhibitor with both *in vitro* and *in vivo* anticancer activities [1, 25, 26, 44–54]. TrxR catalyzes the NADPH-dependent reduction of oxidized thioredoxin and plays a central role in regulating cellular redox homeostasis, cell growth, and apoptosis. Increasing evidence shows that TrxR is over-expressed or constitutively active in many tumor cells. Moreover, TrxR appears to contribute to increased tumor cell growth and a resistance to chemotherapy. Others have proposed that auranofin might be a selective inhibitor of the oncogenic protein kinase C iota (PKCι) protein [55], but others have yet to confirm.

As previously reported, Ridaura^®^ was first developed for the management of rheumatoid arthritis as a disease-modifying anti-rheumatic drug (DMARD) [56]. However, the adverse effects and the concerns about the long-term consequences of the immunosuppression induced by Ridaura^®^ led to the decline in its use [57]. Although, auranofin has shown superior activity in *in vitro* screens across several tumor types [49, 58–61], we noted that it was not effective in other animal xenograft models by our group when given orally (Ma, Pessetto, and Godwin, unpublished data). The only *in vivo* efficacy studies using auranofin that showed activity evaluate the drug as a suspension/solution delivered i.p. [58, 62], but neither studies performed formulations, dose tolerance, or pharmacokinetics studies. Therefore, we sought to determine if altering the route of delivery could replicate the dramatic *in vitro* anticancer activity and improve its efficacy *in vivo*. Previous studies have shown that orally delivered auranofin is rapidly metabolized and intact auranofin has never been detected in the blood [63]. Thus, studies of the pharmacokinetics of auranofin have involved measurement of gold concentrations. Upon oral administration, 25% of auranofin gets absorbed [18, 64]. The mean terminal plasma half-life of auranofin gold at steady state in previous studies was 26 days (range 21 to 31 days; *n* = 5). The mean terminal body half-life was 80 days (range 42 to 128; *n* = 5). About 60% of the absorbed gold (15% of the administered dose) from a single dose of auranofin is excreted in urine; the remaining gold is excreted in feces [18]. To more closely mimic the *in vitro* activity of auranofin, we evaluated alternative routes of delivery and demonstrated that the amount of gold from the i.p. suspension dose is 4 to 5 times that obtained by the oral route.

Auranofin has been evaluated in the several cancer clinical trials (clinicaltrials.gov). A clinical proof of concept trial, evaluating auranofin as a single agent for the treatment of chronic lymphocytic leukemia (CLL) was recently completed (clinicaltrials.gov, NCT01419691) by University of Kansas Medical Center. Based on the initial results in CLL, a multi-center clinical proof of concept trial is currently being initiated to evaluate auranofin in combination with bortezomib for the treatment of Mantle Cell Lymphoma (Weir, personal communications). In a recent clinical study, Jatoi and colleagues reported that 10 asymptomatic ovarian cancer patients with elevated CA-125 received 3 mg of auranofin orally twice per day and were evaluated for up to ~6 months. Although well tolerated, oral delivery of auranofin had little effect or no effect on improving progress-free survival; the median PFS was 2.8 months (95% CI: 1.3–3.8) [65], further suggesting our idea that the gold levels obtained via oral delivery may be insufficient to achieve its optimal anti-cancer effects.

The other class of drugs identified in our study is HSP90 inhibitors. HSP90 is a highly conserved molecular chaperone protein that aids in the stability and proper folding of client proteins. It is vital for cell transformation, proliferation, and survival. Client proteins that are dependent on HSP90 were identified in cancer hallmarks such as growth and evasion of apoptosis. Thus, HSP90 is considered as a potential target for cancer therapy [66, 67]. Our validation screen identified three HSP90 inhibitors: geldanamycin, ganetespib, and AUY922. Geldanamycin, a benzoquinone ansamycin with antibiotic and antitumor activities, was one of earliest HSP90 inhibitors [68, 69]. The dependence on NAD(P)H:quinone oxidoreductase and the gastrointestinal and hepatic toxicities limited geldanamycin and its derivatives’ clinical use. Ganetespib and AUY922 emerged as second-generation, non-geldanamycin HSP90 inhibitors with better pharmacokinetics and safety profiles [70, 71]. Ganetespib inhibits HSP90 protein by acting on the ATP-binding domain at the N-terminus and has shown a preclinical antitumor activity in several solid and hematological tumors [17, 67, 68, 72, 73]. AUY922 is an isoxazole-based HSP90 inhibitor that antagonizes the ATPase activity of the protein. It has shown *in vitro* and *in vivo* potent antitumor activity [72, 73]. Thirty eight studies found for ganetespib on CllinicalTrials.gov. Two trials were terminated with results as of July 20, 2016 (NCT01798485 and NCT01227018).

Even though the NCT01798485 trial showed no safety issues with ganetespib, Synta suspended the phase 3 study of ganetespib in combination with docetaxel versus docetaxel alone in patients with advanced non-small-cell lung cancer. NCT01227018, a phase 2 study STA-9090 as second or third-line therapy for metastatic pancreas cancer, was terminated due to interim analysis found the study drug to be ineffective (15 patients were enrolled). Three trials are recruiting patients with small cell lung cancer, epithelial ovarian cancer, fallopian tube cancer, primary peritoneal cancer, and breast cancer.

In the *in vivo* study, auranofin via i.p. delivery, ganetespib, and the combination treatments significantly increased the survival rate of the mice. The new route of delivery of auranofin alone or in combination with ganetespib did not show any noticeable toxicity or adverse effects, except for a tail lesion in two mice at the site of the i.v. injection of ganetespib. In all, auranofin, an FDA-approved drug for the oral treatment of rheumatoid arthritis, and ganetespib, an investigational HSP90 inhibitor, were predicted to have activity in EWS. Activity was subsequently confirmed *in vitro* via primary and secondary screens. *In vivo* proof of principle was demonstrated in a validated xenograft mouse model of EWS. When given in combination, auranofin and ganetespib were synergistic; however, reformulation of auranofin was required to achieve anti-cancer levels of the gold particle, suggesting caution when repurposing certain drugs for cancer therapies, *i.e*., the potential need to “repurpose” the formulation and delivery of a repurposed drug for cancer therapy. Efforts are underway to translate these findings to a clinical proof of concept study in EWS patients with this debilitating and often-fatal disease.

## MATERIALS AND METHODS

### Compound library

The FDA-approved drug library was provided by the Lead Development and Optimization Shared Resource (LDOSR) within the NCI Cancer Center at the University of Kansas. The library contains 2,316 FDA-approved drugs (1,536 unique chemical entities) with known bioavailability and safety profile in humans. Drugs were present at a concentration of 10 mmol/L in DMSO. For validation, auranofin was synthesized at the University of Kansas and provided by LDOSR with a purity of 100%. Ganetespib was gifted by Synta Pharmaceuticals (Dr. David Proia). Other drugs were purchased from SelleckChem.

### Cell culture

The A673 cell line, an EWS cell line [74], was kindly provided by Dr. Mizuki Azuma (University of Kansas, Lawrence, KS). A673 cells were cultured in Dulbecco's modified Eagle's medium supplemented with 2 mM L-glutamine and 10% fetal bovine serum (FBS). TC-71, COG-E-352 and CHLA-258 cell lines were obtained from Children's Oncology Group Cell Culture and the Xenograft Repository (Texas Tech University Health Sciences Center, TX). TC-71, COG-E-352, and CHLA-258 cell lines were maintained in Iscove's Modified Dulbecco's Medium supplemented with 20% FBS, 2 mM L-glutamine and 1XITS (American Type Culture Collection, ATCC, Manassas, VA). RD-ES (cultured in RPMI-1640 medium with 15% FBS), SK-ES-1 (cultured in McCoy's 5A medium with 10% FBS) cell lines were purchased from ATCC. Hs 822.T and Hs 863.T (both purported to be derived from EWS), and Hs 919.T (derived from an osteoid tumor) were also purchased from ATCC and were cultured in Dulbecco's Modified Eagle's Medium with 10% FBS. All cell lines were supplemented with 1% penicillin/streptomycin and were maintained in a 5% CO_2_ atmosphere at 37°C. The EWS fusion status of each cell line is shown below (Table 1).

### *In silico* prediction

Three *in silico* approaches (disease-based, siRNA-based, resistance-based approach) were used to predict drugs for EWS. The workflow is shown in Figure 1 and the details of the methods are described in the supplementary material. Briefly, first, the drugs that are predicted to reverse disease gene expression were identified. The disease gene expression signature was created as the overlap of the signatures from two meta-analysis studies [75, 76]. Next, since the EWSR1/FLI1 fusion gene is thought to be a causative mutation in most EWS, we searched for drugs that possess the gene expression profile similar to the silence of EWS/FLI1. The siRNA of EWS/FLI1 (siEWS/FLI1) mediated signature was obtained from the previous study [77]. Lastly, since a large number of EWS patients are resistant to chemotherapy, we searched for drugs that were predicted to sensitize drug resistance. The drug resistance signature was computed by comparing the pre-treatment samples of patients responding to chemotherapy and those responding poorly to chemotherapy [78]. Drug expression signature database was built from the Connectivity Map (CMap) [79] and part of the Library of Integrated Cellular Signatures (LINCS, http://lincscloud.org). The drug signatures that are not robust across multiple experiments were removed. The comparison of disease signatures and drug signatures was described previously [13]. The negative score shows the drug signature is anti-correlated with the disease signature and the positive score shows the drug signature is correlated with the disease signature. A false discovery rate (FDR) value of less than 0.05 was used to select hits and only the top 20 hits in each approach were selected for *in vitro* validation. The drug hits predicted from the three approaches were merged. We further manually selected drugs for *in vitro* validation based on their availability in the *in vitro* screening library, novelty and potential side effects.

### *In vitro* high-throughput screening

Drugs or vehicle (DMSO) were preloaded by the LDOSR as 250 nL aliquots on an Echo550 platform to each well to give a final concentration of 1 μmol/L in a total of 25 μL. Cells were grown to 80–90% confluence, harvested and aliquoted into 384-well plates (black μClear microplates, Greiner bio-one, Germany) at concentrations of 750–1,500 cells per well in a total volume of 25 μl/well using a Matrix Wellmate (Thermo Scientific). Cells were cultured for 72 hours in a 5% CO_2_ atmosphere at 37°C. Aliquots of 25 μl CellTiter-Glo^®^ Reagent (Promega) were added directly to each well, the plates were incubated at room temperature for 20 min and the luminescence signal was measured according to the manufacturer's protocol. The measurements were made using Infinite^®^ M200 Pro plate reader (Tecan, Switzerland). Performance of the assay was calculated and the Z’ factors were ≥ 0.5 [80]. Data were normalized to percentage inhibition. Samples exhibiting > 50% growth inhibition in the presence of 1 μmol/L drug were classified as positive hits.

### Hits validation and combination study

For hits validation and combination study, cells were harvested and aliquoted into 384-well plates at concentrations of 750–1,500 cells per well in a total of 20 μl/well using a Matrix Wellmate (Thermo Scientific). Five μl of culture media containing either vehicles or drug were added to each well the next day (drug concentrations range from 1 nmol/L to 50 μmol/L). For combination study, selected drugs were archived in robotically accessible plates, to which media was added in preparation for addition to master plates by a Nimbus 96 liquid-dispensing workstation (Hamilton). Liquid transfers to dilution and assay plates were handled using the same workstation. Each 384-well master plate contained four 9 by 9 dose-matrix blocks, with eight serial two-fold dilutions (concentrations range from 7.8 nmol/L to 1 μmol/L). Additional wells were reserved for untreated, and vehicle-treated control wells. Cell proliferation was evaluated using Cell-Titer Glo^®^ reagents (Promega). IC_50_ values were determined for each drug using SigmaPlot (Systat Software). The combination index values were calculated using SigmaPlot (Systat Software) and CompuSyn [25, 81–83].

### Auranofin and ganetespib formulations

A parenteral suspension formulation was prepared to support IP administration of auranofin in EWS mouse xenograft studies. The injectable suspension formulation contained 0.67 mg/ml of auranofin with 0.5% Hydroxypropyl methylcellulose (HPMC, viscosity grade K4M) in 5% dextrose in water for injection (D5W). Ganetespib was administered using an injectable solution formulation (20% Cremophor RH40 and 80% D5W) provided by Synta Pharmaceuticals.

### Auranofin dose tolerance studies

The auranofin dose tolerance and pharmacokinetics study protocols were approved by the KU-Lawrence Institutional Animal Care and Use Committee (KU-IACUC). Subchronic dose tolerance studies were conducted in BALB/c mice to support the design of EWS mouse xenograft studies. By establishing the Maximum Tolerated Dose (MTD), we defined the upper limit of doses selected for EWS mouse xenograft and *in vivo* pharmacokinetics studies. Mice received a single dose of auranofin solution and suspension administered by i.v. and i.p. routes of administration. Following a starting dose of 1 mg/kg, doses were escalated in subsequent mice until intolerable drug-related adverse effects were observed. MTD was defined as the maximum dose that was not associated dose-limiting adverse effects in mice. The MTD was confirmed in a group of 3 mice. Mice were continuously monitored for up to 4 hours post-dose.

### Auranofin pharmacokinetics

The single-dose plasma pharmacokinetics of gold were characterized in 36 BALB/c mice following administration of a single 12 mg/kg i.p. suspension dose of auranofin. The mice were pretreated 24 hours prior to auranofin, with a single 150 mg/kg i.v. dose of ganetespib. Blood samples for determination of blood gold concentration were obtained prior to, and at 5, 15, 30 and 60 minutes, 2, 4, 8, 12, 24 and 48 hours following administration of auranofin. In addition, a trough (plateau) blood sample, collected at 24 hours post-dose, was obtained in mice following the fifth of five consecutive 12 mg/kg once daily i.p. doses of auranofin suspension. Blood samples were collected from each animal via cardiac puncture. Whole blood aliquots of 500 μL obtained from each of the three animals per time point were assayed using a validated inductively coupled plasma mass spectrometry (ICPMS) method to quantify blood gold concentration. Mean blood gold concentrations were determined for each blood collection time point. Non-parametric pharmacokinetic data analysis was performed on the resultant mean blood gold concentration-time data using WinNonLin^®^, Version 6.2 (Centara USA, Inc, St. Louis, MO).

### *In vivo* xenograft mouse model

The guidelines adopted by the Institutional Animal Care and Use Committee of the University of Kansas Medical Center (KUMC-IACUC) were followed in all performed procedures. All experimental protocols were approved by KUMC-IACUC. Homozygous athymic nude (Foxn1nu/Foxn1nu) female mice at the age of 4-weeks were purchased from Jackson Laboratories. After one week, one million A673 cells in PBS (pH = 7.3) were injected into mice. The cells were injected intramuscularly proximal to the tibia and treatment started after 15 days of inoculation. Mice developing palpable tumors were divided into 4 arms and treated for up to 18 days: vehicle (20% Cremophor RH40 and 80% D5W iv and 0.5% HPMC K4M ip), auranofin (12 mg/kg i.p. injection 5 days per week), ganetespib (150 mg/kg i.v. injection once weekly), as well as auranofin and ganetespib combined. Mice were evaluated for overall health and weighed every week. When tumor size impeded mobility, mice were sacrificed and necropsies were performed. Tumors were then dissected, measured and processed for analysis. The liver, kidneys, and spleen were also snap frozen in liquid nitrogen or fixed in 10% neutral buffered formalin and paraffin embedded for toxicity studies. Tissues were then subjected to hematoxylin and eosin (H&E) staining and immunohistochemical staining. The immunohistochemical staining method and antibodies used in the study were listed in the supplementary data.

### Data analysis and statistics

*In vitro* data were reported as mean ± SD of 3 independent experiments. In the statistical analyses for the *in vivo* xenograft study, Kaplan-Meier survival curves were used to determine the difference in survival among the treatment groups. A *p-value* less than 0.05 was considered as statistically significant. All statistical analyses were performed using SigmaPlot 12.3 (Systat Software, Inc. CA, USE).

## SUPPLEMENTARY MATERIALS FIGURES






