# Supplementary material for: In silico and in vitro drug screening identifies new therapeutic approaches for Ewing sarcoma
Source: Oncotarget. 2016 Nov 16;8(3):4079–95. doi: 10.18632/oncotarget.13385 (PMC5354814; doi:10.18632/oncotarget.13385)
Supplement: Supplementary file 1 [file oncotarget-08-4079-s001.pdf]

In silico and *in vitro* drug screening identifies new therapeutic approaches for Ewing sarcoma

Supplementary Materials

SUPPLEMENTARY METHODS

Disease signatures

An increasing number of gene expression data of EWS samples have been profiled and published. Because the cell of origin of EWS is unknown, many studies have relied on heterologous cell types to study the fusion protein, resulting in multiple microarray experiments. By comparative analysis of different studies, Hancock *et al*. identified a conserved core EWS-FLI signature [1]. Kauer *et al.* [2] compared the primary EWS tumor samples with 80 normal tissues and found that the data using mesenchymal progenitor cell (MPC) as the reference tissue is highly anti-correlated with the data by the knockdown of EWS-FLI1 in a panel of five EWS related cell lines. Using MPC as a reference tissue, they constructed a molecular function map of EWS. We compiled 783 and 619 differentially expressed genes from Hancock's study and Kauer's study, respectively. 174 genes (including 122 up, 52 down regulated genes) were shared by the two studies. We used the fold change from the Kauer's study. The mean was used to merge the fold change of the gene with multiple probes.

siEWS/FLI1 mediated signature

EWS is characterized by the presence of specific gene fusions which most frequently involve the EWS gene on chromosome 22 and FLI-1 gene on chromosome 11. Loss of expression of this fusion gene resulted in the complete arrest of growth. Prieur *et al.* [3] performed microarray after the knockdown of EWS/FLI1 using siRNA reagent and found 86 differentially expressed. 76 (71 up and 5 down-regulated genes) of them were mapped to the genes profiled in CMap. Since their finding led to the discovery of IGFBP-3 as the therapeutic target in EWS, we built the siEWS/FLI1 signature using their published results.

Drug resistance signature

Schaefer *et al.* profiled the global gene expression of 27 primary samples of the Ewing's Sarcoma family of tumors prior to chemotherapy. The samples were categorized into two groups: good response group and poor response group. The samples without response data were ignored. Their microarray data were downloaded from ArrayExpress (ID: E-MEXP-1142), followed by quantile normalization. RankProd [4] was used for computing differentially expressed genes between two groups. If the number of up/down genes exceed to 150, then only top 150 genes ranked by fold change were selected. As a result, the drug resistance signature consisting of 140 up and 150 down regulated was created.

Drug gene expression database

We included all instances from the second version of Connectivity Map (CMap) [5]. It includes 6,100 instances consisting of 1,309 drugs (or chemical compounds) tested in three major cell lines. In order to expand drug space, we also added some instances from LINCS (lincscloud.org), into the drug library. Since the development of LINCS was undergoing and no systematic evaluation of this dataset was published when we started to use LINCS (as of Jan 2013), we decided to take part of drug signatures as test cases in this work. We queried LINCS database from their website using a disease gene expression signature and retrieved drug gene expression data via the API. Since LINCS has over 1 million signatures, retrieving the expression data of all the compounds was not possible. The batch downloading of the signatures was not available at that time. We thus only selected the top scored drug hits where the compounds were treated in 6 hours (a treatment duration primarily used in CMap). Then we merged the drug gene expression into the CMap library. The probes of each instance were ranked by their expression value (z score), and each probe was then annotated using Entrez Gene id, an identifier used in CMap. For the gene id with multiple probes, its rank was an average of the ranks of its probes. For the gene id in CMap that was not profiled in LINCS, its rank was replaced with the median of the ranks of all mapped genes. In the end, we added 656 instances consisting of 26 compounds into the CMap library. In addition, we only kept the drug instances where its profile is correlated to at least one profile of the same drug ($p < 0.05$). In total, 3093 instances, consisting of 700 drugs, were used for prediction.

Predict drugs using three computational approaches

Disease-based approach: We used the disease signature to query the drug expression database and selected the top 20 negatively scored hits. siRNA-based approach: We used the siEWS-FLI1 mediated signature to query the drug expression database and selected the top 20 positively scored hits. Resistance-based signature: We used the drug-resistance signature to query the drug expression database and select the top 20 negatively scored hits.

siRNA transfection followed by auranofin treatment

A673 type I Ewing cells were plated at a density of 8×10^4 cells per well in 6-well plates in DMEM supplemented with 10% FBS. The cells were transfected 24 h later with 50 nmol/L of two *EWS-FLI1* targeting siRNAs respectively (siFLI1-#3, 5′-AGUCGUCCAUGU ACAAGUA-3′; siBPEF1, 5′-GCAGCAGAACCCUUCU UAU-3′) or non-targeting control siRNA (siControl, 5′-UAAGGCUAUGAAGAGAUAC-3′) using DharmaFECT 1 Transfection Reagent (Dharmacon, Lafayette, CO) according to the manufacturer's instructions. All siRNAs were purchased from Dharmacon (Lafayette, CO). Forty-eight hours after transfection, cells were washed and harvested by trypsinization. A portion of A673 cells were seeded at a density of 2×10^3 cells per well in a 96-well plate, grown for 24 h and then subjected to two-fold serial dilutions of auranofin ranging from 7.8 nmol/L to 1 μmol/L. The remaining cells were used to detect the expression of EWS-FLI1 after siRNA transfection by using western blot analysis. After 72 h of auranofin treatment, cell viability was evaluated using the CellTiter-Blue assay (Promega, Madison, WI) according to the manufacturer's instructions and the fluorescent signal was read using Infinite® M200 Pro plate reader (Tecan, Switzerland). IC_{50} values were determined by using GraphPad Prism 5 software (La Jolla, CA).

Western blot analysis

Forty-eight hours after siRNA transfection, cells were washed with PBS and then lysed in ice cold RIPA buffer (ThermoFisher Scientific, Waltham, MA) supplemented with cOmplete™ Mini Protease Inhibitor Cocktail Tablets (Sigma, St. Louis, MO). Protein concentration was determined by using the Bradford Protein Assay Kit (Bio-Rad, Hercules, CA). 60 μg of proteins were resolved on the 4–20% Mini-PROTEAN TGX™ Precast Gels (Bio-Rad, Hercules, CA) and transferred onto PVDF membranes (Bio-Rad, Hercules, CA). Immunoblotting was done with the following antibodies: rabbit polyclonal anti-FLI1 (C-19) (Santa Cruz Biotechnology, Dallas, TX), mouse monoclonal anti-β-actin (Sigma, St. Louis, MO), HRP conjugated goat anti-rabbit IgG (Cell Signaling, Danvers, MA) and HRP conjugated horse anti-mouse IgG (Cell Signaling, Danvers, MA). Membranes were developed using ECL 2 Western Blotting Substrate (ThermoFisher Scientific, Waltham, MA).

REFERENCES

1. Hancock JD, Lessnick SL. A transcriptional profiling meta-analysis reveals a core EWS-FLI gene expression signature. Cell Cycle. 2008; 7:250–6.
2. Kauer M, et al. A molecular function map of Ewing's sarcoma. PLoS One. 2009; 4:e5415.
3. Prieur A, et al. EWS/FLI-1 silencing and gene profiling of Ewing cells reveal downstream oncogenic pathways and a crucial role for repression of insulin-like growth factor binding protein 3. Mol Cell Biol. 2004; 24:7275–83.
4. Hong F, et al. RankProd: a bioconductor package for detecting differentially expressed genes in meta-analysis. Bioinformatics. 2006; 22:2825–7.
5. Lamb J, et al. The Connectivity Map: using gene-expression signatures to connect small molecules, genes, and disease. Science. 2006; 313:1929–1935.

Supplementary Material Reference

18. Inc. PL. RIDAURA® (auranofin) Capsules. 2011. (See Supplementary Material Reference 18).
19. Inc. XP. RIDAURA® Auranofin Capsules 3 mg product monograph. Retrieved from http://www.xediton.com/RIDAURA_PRODUCT%20MONOGRAPH.pdf. 2010. See Supplementary Material Reference 19.

Supplementary Table S1: Automation protocol for drug repurposing screen

Step	Event	Parameter	Description	Notes
**1**	Pre-spot library compoundsor DMSO	250 nl	1 µmol/µl	Instrument: Echo 550
**2**	Add reagent	20 µl	Cells: 750–1,500 cells/ well	Instrument: Matrix Wellmate
**3**	Incubate	72 hrs	5% CO_2/37°C incubation	
**4**	Add reagent	25 µl	Promega CellTiter-Glo	
**5**	Incubate	20 min	Room temperature	
**6**	Read	Luminescence		Instrument: Infinite M200 Pro

Supplementary Table S2: Summary of PK/PD studies of auranofin

Formula	Dose/Route	Cmax (µg/L)	Tmax (hr)	Half Life (hr)	AUC (0 to ∞) (hr*µg/L)
Solution	Single 4 mg/kg IP	4071	1	29.7	210336
Solution	Five daily 4 mg/kg IP	10384			
Suspension	Single 12 mg/kg IP	13068	1	26.4	550846
Suspension	Five daily 12 mg/kg IP	23874			
Suspension	Single 10 mg/kg Oral	3680	24	28.4	187700
Suspension	Five daily 10 mg/kg Oral	5780			

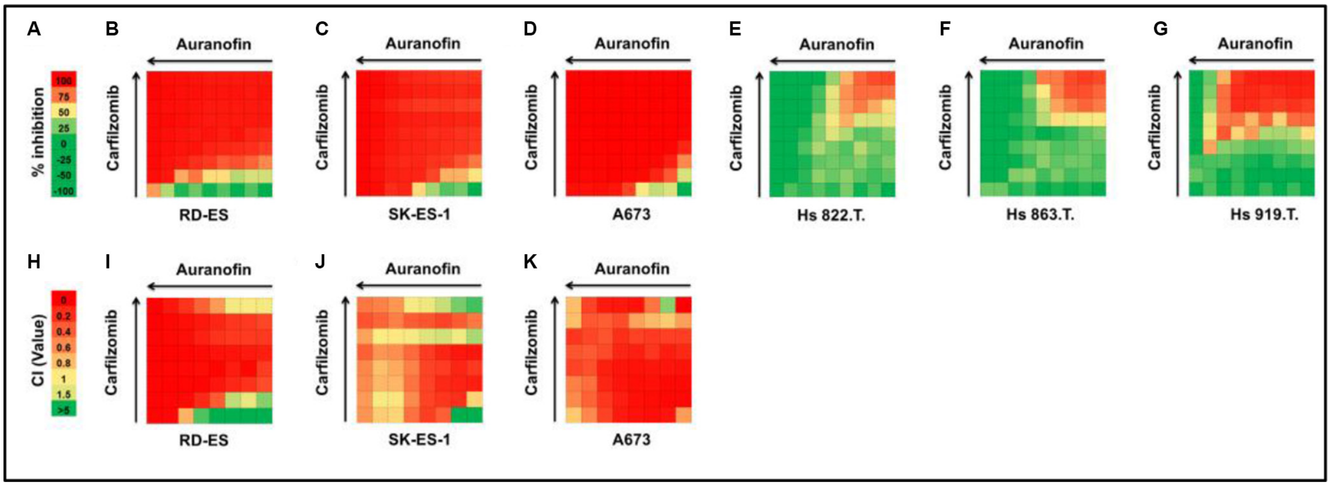

Supplementary Figure S1: Combination analysis for auranofin and carfilzomib in EWS cell lines and three control cell lines (Hs 822.T., Hs 863.T., and Hs 919.T.). (**A**) Color scale for drug inhibition values. (**B–G**) Synergy between auranofin and ganetespib was tested by CellTiter-Glo assay at 64 different drug combinations (auranofin concentration range from 2 µmol/L to 16 nmol/L, carfilzomib concentration range from 1 µmol/L to 8 nmol/L, half-dilutions for each drug) for each cell line. (**H**) Color scale for combination index (CI) values. (**I–K**) CI values. CI value < 1 is considered synergistic. Arrows indicate the increasing concentration of each drug.

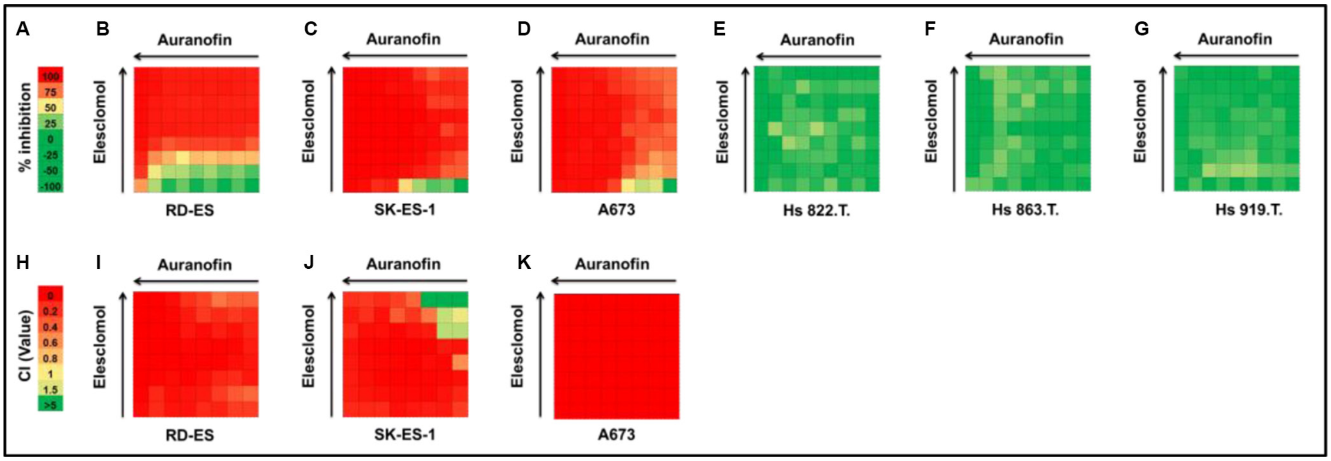

Supplementary Figure S2: Combination analysis for auranofin and elesclomol in EWS cell lines and three control cell lines (Hs 822.T., Hs 863.T., and Hs 919.T.). (**A**) Color scale for drug inhibition values. (**B**–**G**) Synergy between auranofin and elesclomol was tested by CellTiter-Glo assay at 64 different drug combinations (auranofin concentration range from 2 µmol/L to 16 nmol/L, elesclomol concentration range from 2 µmol/L to 16 nmol/L, half-dilutions for each drug) for each cell line. (**H**) Color scale for combination index (CI) values. (**I**–**K**) CI values. CI value < 1 is considered synergistic. Arrows indicate the increasing concentration of each drug.

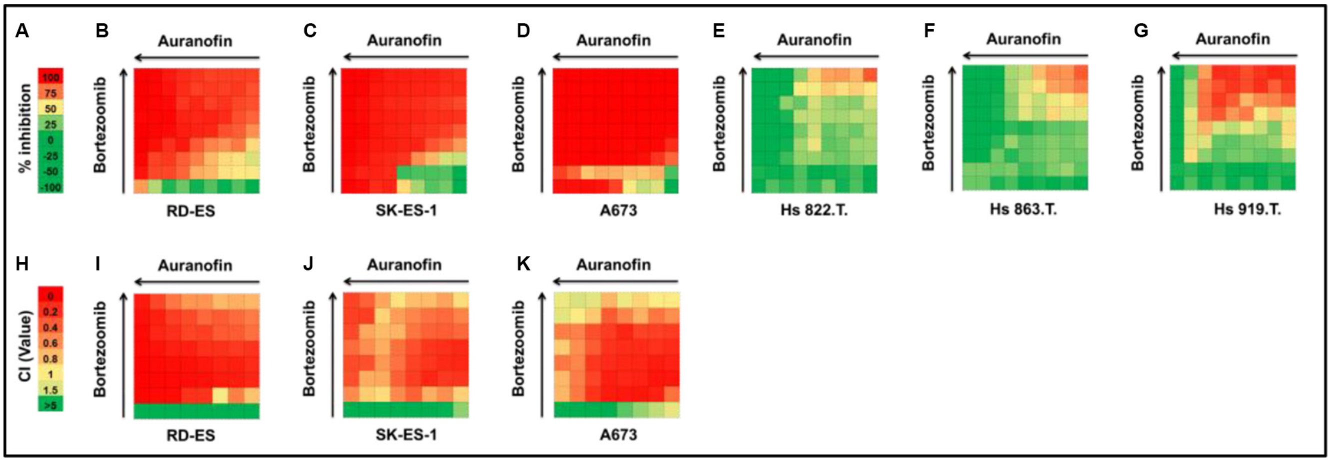

Supplementary Figure S3: Combination analysis for auranofin and bortezomib in EWS cell lines and three control cell lines (Hs 822.T., Hs 863.T., and Hs 919.T.). (**A**) Color scale for drug inhibition values. (**B**–**G**) Synergy between auranofin and bortezomib was tested by CellTiter-Glo assay at 64 different drug combinations (auranofin concentration range from 2 µmol/L to 16 nmol/L, bortezomib concentration range from 1 µmol/L to 8 nmol/L, half-dilutions for each drug) for each cell line. (**H**) Color scale for combination index (CI) values. (**I**–**K**) CI values. CI value < 1 is considered synergistic. Arrows indicate the increasing concentration of each drug.

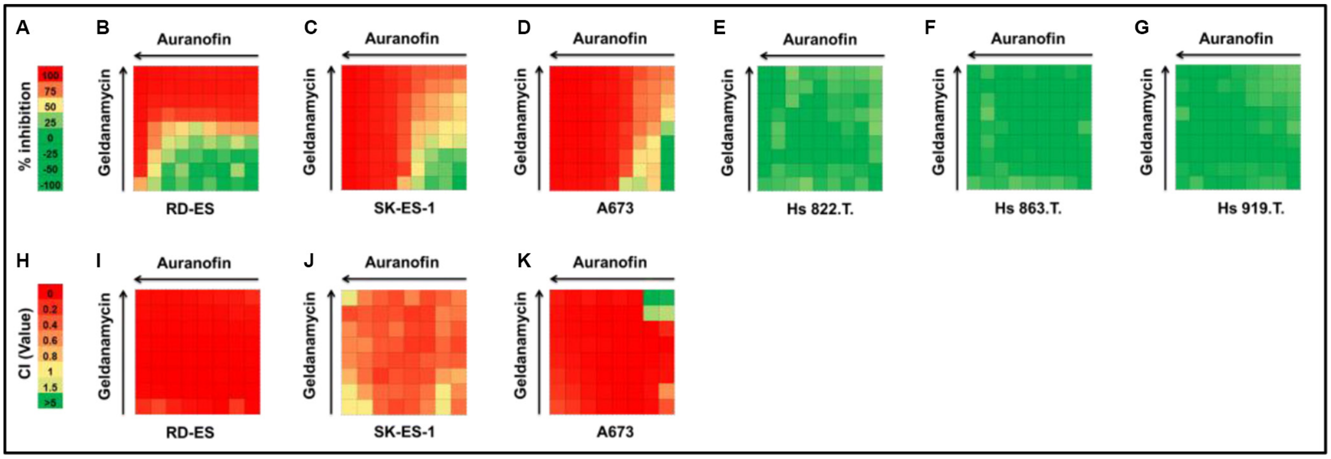

Supplementary Figure S4: Combination analysis for auranofin and geldanamycin in EWS cell lines and three control cell lines (Hs 822.T., Hs 863.T., and Hs 919.T.). (**A**) Color scale for drug inhibition values. (**B–G**) Synergy between auranofin and geldanamycin was tested by CellTiter-Glo assay at 64 different drug combinations (auranofin concentration range from 2 µmol/L to 16 nmol/L, geldanamycin concentration range from 1 µmol/L to 8 nmol/L, half-dilutions for each drug) for each cell line. (**H**) Color scale for combination index (CI) values. (**I–K**) CI values. CI value < 1 is considered synergistic. Arrows indicate the increasing concentration of each drug.

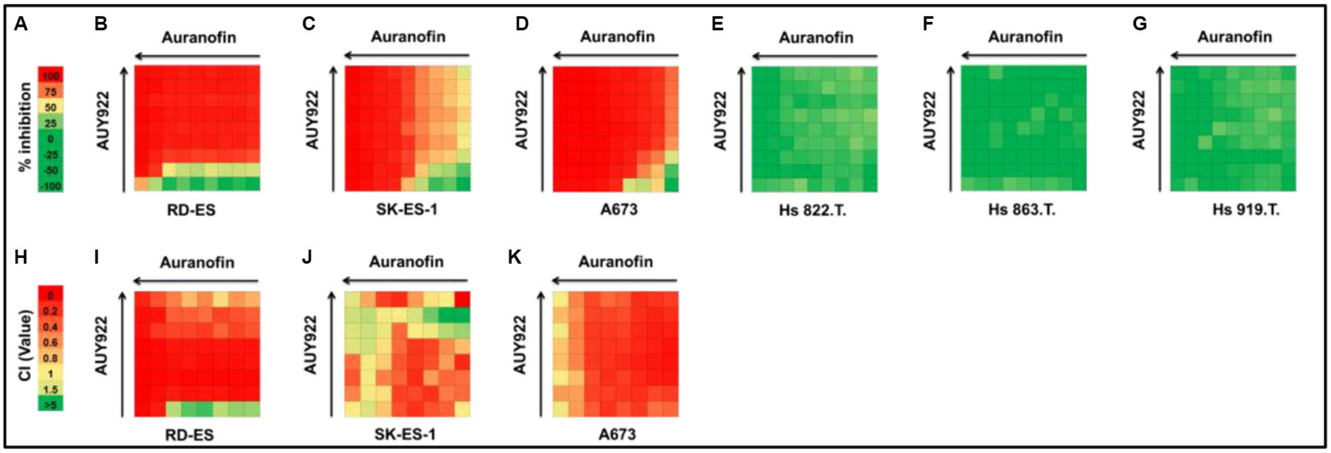

Supplementary Figure S5: Combination analysis for auranofin and AUY922 in EWS cell lines and three control cell lines (Hs 822.T., Hs 863.T., and Hs 919.T.). (**A**) Color scale for drug inhibition values. (**B–G**) Synergy between auranofin and AUY922 was tested by CellTiter-Glo assay at 64 different drug combinations (auranofin concentration range from 2 µmol/L to 16 nmol/L, AUY922 concentration range from 1 µmol/L to 8 nmol/L, half-dilutions for each drug) for each cell line. (**H**) Color scale for combination index (CI) values. (**I–K**) CI values. CI value < 1 is considered synergistic. Arrows indicate the increasing concentration of each drug.

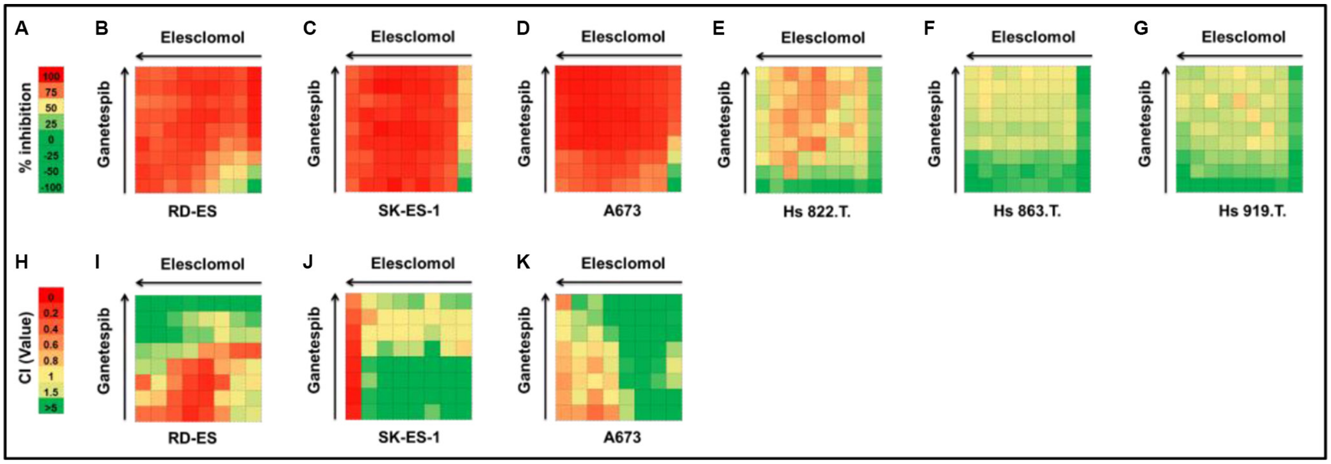

Supplementary Figure S6: Combination analysis for elesclomol and ganetespib in EWS cell lines and three control cell lines (Hs 822.T., Hs 863.T., and Hs 919.T.). (**A**) Color scale for drug inhibition values. (**B–G**) Synergy between elesclomol and ganetespib was tested by CellTiter-Glo assay at 64 different drug combinations (elesclomol concentration range from 2 µmol/L to 16 nmol/L, ganetespib concentration range from 1 µmol/L to 8 nmol/L, half-dilutions for each drug) for each cell line. (**H**) Color scale for combination index (CI) values. (**I–K**) CI values. CI value < 1 is considered synergistic. Arrows indicate the increasing concentration of each drug.

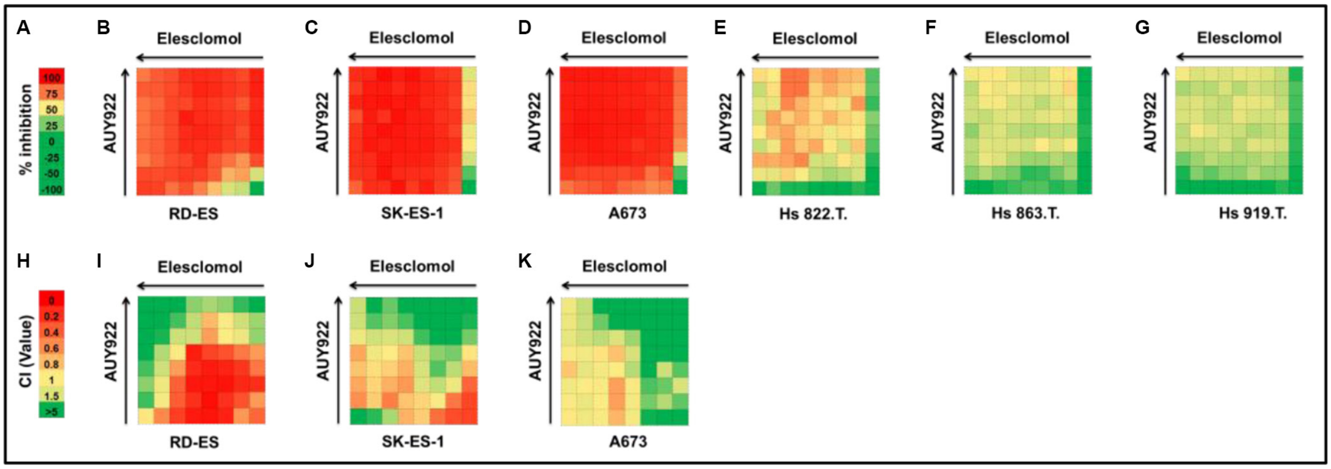

Supplementary Figure S7: Combination analysis for elesclomol and AUY922 in EWS cell lines and three control cell lines (Hs 822.T., Hs 863.T., and Hs 919.T.). (**A**) Color scale for drug inhibition values. (**B–G**) Synergy between elesclomol and AUY922 was tested by CellTiter-Glo assay at 64 different drug combinations (elesclomol concentration range from 2 µmol/L to 16 nmol/L, AUY922 concentration range from 1 µmol/L to 8 nmol/L, half-dilutions for each drug) for each cell line. (**H**) Color scale for combination index (CI) values. (**I–K**) CI values. CI value < 1 is considered synergistic. Arrows indicate the increasing concentration of each drug.

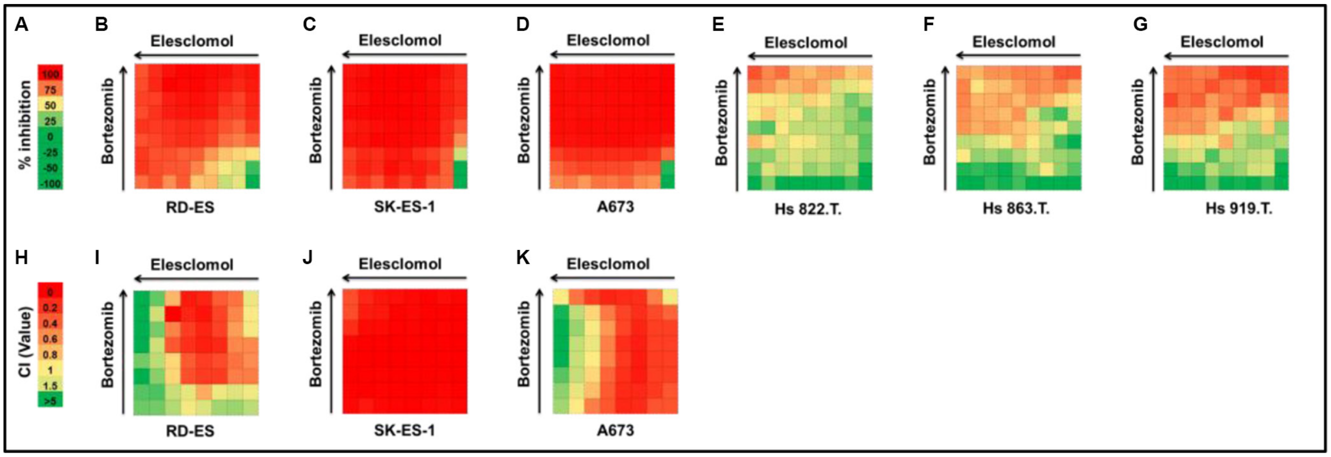

Supplementary Figure S8: Combination analysis for elesclomol and bortezomib in EWS cell lines and three control cell lines (Hs 822.T., Hs 863.T., and Hs 919.T.). (**A**) Color scale for drug inhibition values. (**B–G**) Synergy between elesclomol and bortezomib was tested by CellTiter-Glo assay at 64 different drug combinations (elesclomol concentration range from 2 µmol/L to 16 nmol/L, bortezomib concentration range from 1 µmol/L to 8 nmol/L, half-dilutions for each drug) for each cell line. (**H**) Color scale for combination index (CI) values. (**I–K**) CI values. CI value < 1 is considered synergistic. Arrows indicate the increasing concentration of each drug.

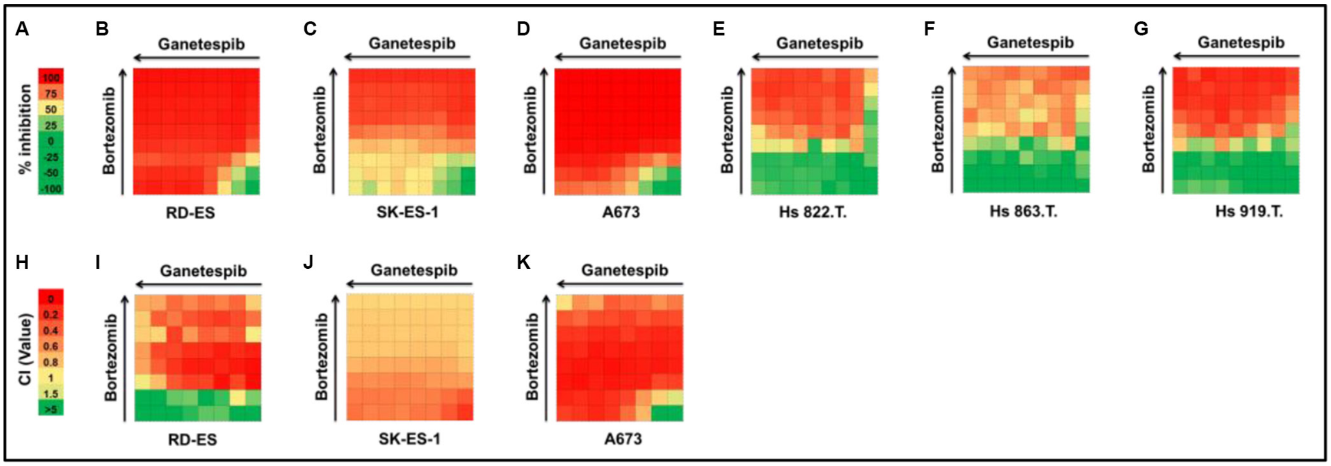

Supplementary Figure S9: Combination analysis for ganetespib and bortezomib in EWS cell lines and three control cell lines (Hs 822.T., Hs 863.T., and Hs 919.T.). (**A**) Color scale for drug inhibition values. (**B–G**) Synergy between ganetespib and bortezomib was tested by CellTiter-Glo assay at 64 different drug combinations (ganetespib concentration range from 1 µmol/L to 8 nmol/L, bortezomib concentration range from 1 µmol/L to 8 nmol/L, half-dilutions for each drug) for each cell line. (**H**) Color scale for combination index (CI) values. (**I–K**) CI values. CI value < 1 is considered synergistic. Arrows indicate the increasing concentration of each drug.

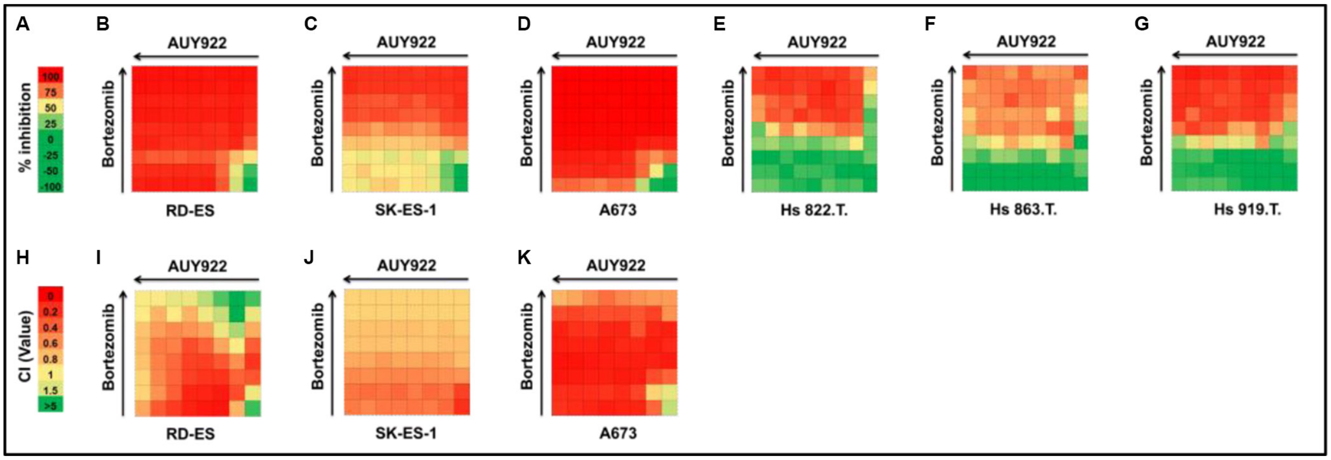

Supplementary Figure S10: Combination analysis for AUY922 and bortezomib in EWS cell lines and three control cell lines (Hs 822.T., Hs 863.T., and Hs 919.T.). (**A**) Color scale for drug inhibition values. (**B**–**G**) Synergy between AUY922 and bortezomib was tested by CellTiter-Glo assay at 64 different drug combinations (AUY922 concentration range from 1 µmol/L to 8 nmol/L, bortezomib concentration range from 1 µmol/L to 8 nmol/L, half-dilutions for each drug) for each cell line. (**H**) Color scale for combination index (CI) values. (**I**–**K**) CI values. CI value < 1 is considered synergistic. Arrows indicate the increasing concentration of each drug.

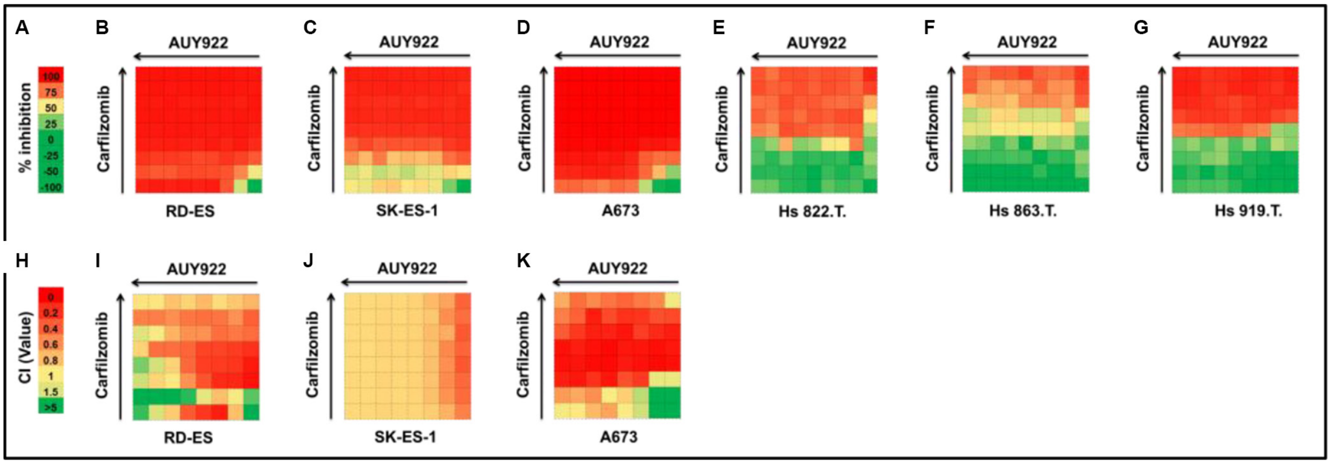

Supplementary Figure S11: Combination analysis for AUY922 and carfilzomib in EWS cell lines and three control cell lines (Hs 822.T., Hs 863.T., and Hs 919.T.). (**A**) Color scale for drug inhibition values. (**B**–**G**) Synergy between AUY922 and carfilzomib was tested by CellTiter-Glo assay at 64 different drug combinations (AUY922 concentration range from 1 µmol/L to 8 nmol/L, carfilzomib concentration range from 1 µmol/L to 8 nmol/L, half-dilutions for each drug) for each cell line. (**H**) Color scale for combination index (CI) values. (**I**–**K**) CI values. CI value < 1 is considered synergistic. Arrows indicate the increasing concentration of each drug.
