# Supplementary material for: In silico and in vitro drug screening identifies new therapeutic approaches for Ewing sarcoma
Source: Oncotarget. 2016 Nov 16;8(3):4079–95. doi: 10.18632/oncotarget.13385 (PMC5354814; doi:10.18632/oncotarget.13385)
Supplement: Supplementary file 2 [file oncotarget-08-4079-s002.pdf]

PRESCRIBING INFORMATION

RIDAURA®
Auranofin
Capsules

R_x Only

RIDAURA® (auranofin) contains gold and, like other gold-containing drugs, can cause gold toxicity, signs of which include: fall in hemoglobin, leukopenia below 4,000 WBC/cu mm, granulocytes below 1,500/cu mm, decrease in platelets below 150,000/cu mm, proteinuria, hematuria, pruritus, rash, stomatitis or persistent diarrhea. Therefore, the results of recommended laboratory work (See PRECAUTIONS) should be reviewed before writing each RIDAURA prescription. Like other gold preparations, RIDAURA is only indicated for use in selected patients with active rheumatoid arthritis. Physicians planning to use RIDAURA should be experienced with chrysotherapy and should thoroughly familiarize themselves with the toxicity and benefits of RIDAURA.

In addition, the following precautions should be routinely employed:
1. The possibility of adverse reactions should be explained to patients before starting therapy.
2. Patients should be advised to report promptly any symptoms suggesting toxicity. (See PRECAUTIONS—Information for Patients.)

DESCRIPTION
RIDAURA (auranofin) is available in oral form as capsules containing 3 mg auranofin.

Auranofin is (2,3,4,6-tetra-O-acetyl-1-thio-ß-D-glucopyranosato-S-) (triethyl-phosphine) gold. Auranofin contains 29% gold and has the following chemical structure:

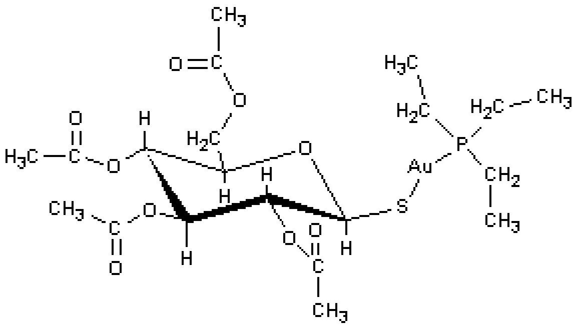

Each RIDAURA capsule, with opaque brown cap and opaque tan body, contains auranofin, 3 mg, and is imprinted with the product name RIDAURA. Inactive ingredients consist of benzyl alcohol, cellulose, cetylpyridinium chloride, D&C Red No. 33, FD&C Blue No. 1, FD&C Red No. 40, FD&C Yellow No. 6, gelatin, lactose, magnesium stearate, povidone, sodium lauryl sulfate, sodium starch glycolate, starch, titanium dioxide and trace amounts of other inactive ingredients.

CLINICAL PHARMACOLOGY
The mechanism of action of RIDAURA (auranofin) is not understood. In patients with adult rheumatoid arthritis, RIDAURA may modify disease activity as manifested by synovitis and associated symptoms, and reflected by laboratory parameters such as ESR. There is no substantial evidence, however, that gold-containing compounds induce remission of rheumatoid arthritis.

Pharmacokinetics: Pharmacokinetic studies were performed in rheumatoid arthritis patients, not in normal volunteers. Auranofin is rapidly metabolized and intact auranofin has never been detected in the blood.

Thus, studies of the pharmacokinetics of auranofin have involved measurement of gold concentrations. Approximately 25% of the gold in auranofin is absorbed.
The mean terminal plasma half-life of auranofin gold at steady state was 26 days (range 21 to 31 days; n=5). The mean terminal body half-life was 80 days (range 42 to 128; n=5). Approximately 60% of the absorbed gold (15% of the administered dose) from a single dose of auranofin is excreted in urine; the remainder is excreted in the feces.
In clinical studies, steady state blood-gold concentrations are achieved in about three months. In patients on 6 mg auranofin/day, mean steady state blood-gold concentrations were 0.68 ±0.45 mcg/mL (n=63 patients). In blood, approximately 40% of auranofin gold is associated with red cells, and 60% associated with serum proteins. In contrast, 99% of injectable gold is associated with serum proteins.
Mean blood-gold concentrations are proportional to dose; however, no correlation between blood-gold concentrations and safety or efficacy has been established.

INDICATIONS AND USAGE
RIDAURA (auranofin) is indicated in the management of adults with active classical or definite rheumatoid arthritis (ARA criteria) who have had an insufficient therapeutic response to, or are intolerant of, an adequate trial of full doses of one or more nonsteroidal anti-inflammatory drugs. RIDAURA should be added to a comprehensive baseline program, including non-drug therapies.
Unlike anti-inflammatory drugs, RIDAURA does not produce an immediate response. Therapeutic effects may be seen after three to four months of treatment, although improvement has not been seen in some patients before six months.
When cartilage and bone damage has already occurred, gold cannot reverse structural damage to joints caused by previous disease. The greatest potential benefit occurs in patients with active synovitis, particularly in its early stage.
In controlled clinical trials comparing RIDAURA with injectable gold, RIDAURA was associated with fewer dropouts due to adverse reactions, while injectable gold was associated with fewer dropouts for inadequate or poor therapeutic effect. Physicians should consider these findings when deciding on the use of RIDAURA in patients who are candidates for chrysotherapy.

CONTRAINDICATIONS
RIDAURA (auranofin) is contraindicated in patients with a history of any of the following gold-induced disorders: anaphylactic reactions, necrotizing enterocolitis, pulmonary fibrosis, exfoliative dermatitis, bone marrow aplasia or other severe hematologic disorders.

WARNINGS
Danger signs of possible gold toxicity include fall in hemoglobin, leukopenia below 4,000 WBC/cu mm, granulocytes below 1,500/cu mm, decrease in platelets below 150,000/cu mm, proteinuria, hematuria, pruritus, rash, stomatitis or persistent diarrhea.
Thrombocytopenia has occurred in 1–3% of patients (See ADVERSE REACTIONS) treated with RIDAURA (auranofin), some of whom developed bleeding. The thrombocytopenia usually appears to be peripheral in origin and is usually reversible upon withdrawal of RIDAURA. Its onset bears no relationship to the duration of RIDAURA therapy and its course may be rapid. While patients' platelet counts should normally be monitored at least monthly (See PRECAUTIONS— Laboratory Tests), the occurrence of a precipitous decline in platelets or a platelet count less than 100,000/cu mm or signs and symptoms (e.g., purpura, ecchymoses or petechiae) suggestive of thrombocytopenia indicates a need to immediately withdraw RIDAURA and other therapies with the potential to cause thrombocytopenia, and to obtain additional platelet counts. No additional RIDAURA should be given unless the thrombocytopenia resolves and further studies show it was not due to gold therapy.
Proteinuria has developed in 3-9% of patients (See ADVERSE REACTIONS) treated with RIDAURA. If clinically significant proteinuria or microscopic hematuria is found (See PRECAUTIONS— Laboratory Tests), RIDAURA and other therapies with the potential to cause proteinuria or microscopic hematuria should be stopped immediately.

PRECAUTIONS

General: The safety of concomitant use of RIDAURA (auranofin) with injectable gold, hydroxychloroquine, penicillamine, immunosuppressive agents (e.g., cyclophosphamide, azathioprine, or methotrexate) or high doses of corticosteroids has not been established.

Medical problems that might affect the signs or symptoms used to detect RIDAURA toxicity should be under control before starting RIDAURA (auranofin).
The potential benefits of using RIDAURA in patients with progressive renal disease, significant hepatocellular disease, inflammatory bowel disease, skin rash or history of bone marrow depression should be weighed against 1) the potential risks of gold toxicity on organ systems previously compromised or with decreased reserve, and 2) the difficulty in quickly detecting and correctly attributing the toxic effect.

The following adverse reactions have been reported with the use of gold preparations and require modification of RIDAURA treatment or additional monitoring. See ADVERSE REACTIONS for the approximate incidence of those reactions specifically reported with RIDAURA.

Gastrointestinal Reactions: Gastrointestinal reactions reported with gold therapy include diarrhea/loose stools, nausea, vomiting, anorexia and abdominal cramps. The most common reaction to RIDAURA is diarrhea/ loose stools reported in approximately 50% of the patients. This is generally manageable by reducing the dosage (e.g., from 6 mg daily to 3 mg) and in only 6% of the patients is it necessary to discontinue RIDAURA (auranofin) permanently. Ulcerative enterocolitis is a rare serious gold reaction. Therefore, patients with gastrointestinal symptoms should be monitored for the appearance of gastrointestinal bleeding.

Cutaneous Reactions: Dermatitis is the most common reaction to injectable gold therapy and the second most common reaction to RIDAURA. *Any eruption, especially if pruritic, that develops during treatment should be considered a gold reaction until proven otherwise.* Pruritus often exists before dermatitis becomes apparent, and therefore should be considered to be a warning signal of a cutaneous reaction. Gold dermatitis may be aggravated by exposure to sunlight or an actinic rash may develop. The most serious form of cutaneous reaction reported with injectable gold is generalized exfoliative dermatitis.

Mucous Membrane Reactions: Stomatitis, another common gold reaction, may be manifested by shallow ulcers on the buccal membranes, on the borders of the tongue, and on the palate or in the pharynx. Stomatitis may occur as the only adverse reaction or with a dermatitis. Sometimes diffuse glossitis or gingivitis develops. A metallic taste may precede these oral mucous membrane reactions and should be considered a warning signal.

Renal Reactions: Gold can produce a nephrotic syndrome or glomerulitis with proteinuria and hematuria. These renal reactions are usually relatively mild and subside completely if recognized early and treatment is discontinued. They may become severe and chronic if treatment is continued after the onset of the reaction. Therefore it is important to perform urinalyses regularly and to discontinue treatment promptly if proteinuria or hematuria develops.

Hematologic Reactions: Blood dyscrasias including leukopenia, granulocytopenia, thrombocytopenia and aplastic anemia have all been reported as reactions to injectable gold and RIDAURA. These reactions may occur separately or in combination at anytime during treatment. Because they have potentially serious consequences, *blood dyscrasias should be constantly watched for through regular monitoring (at least monthly) of the formed elements of the blood throughout treatment.*

Miscellaneous Reactions: Rare reactions attributed to gold include cholestatic jaundice; gold bronchitis and interstitial pneumonitis and fibrosis; peripheral neuropathy; partial or complete hair loss; fever.

Information for Patients: Patients should be advised of the possibility of toxicity from RIDAURA and of the signs and symptoms that they should report promptly. (Patient information sheets are available.)
Women of childbearing potential should be warned of the potential risks of RIDAURA therapy during pregnancy (See PRECAUTIONS— Pregnancy).

Laboratory Tests: CBC with differential, platelet count, urinalysis, and renal and liver function tests should be performed prior to RIDAURA (auranofin) therapy to establish a baseline and to identify any preexisting conditions.
CBC with differential, platelet count and urinalysis should then be monitored at least monthly; other parameters should be monitored as appropriate.

Drug Interactions: In a single patient-report, there is the suggestion that concurrent administration of RIDAURA and phenytoin may have increased phenytoin blood levels.
Carcinogenesis/Mutagenesis: In a 24-month study in rats, animals treated with auranofin at 0.4, 1.0 or 2.5 mg/kg/day orally (3, 8 or 21 times the human dose) or gold sodium thiomalate at 2 or 6 mg/kg injected twice weekly (4 or 12 times the human dose) were compared to untreated control animals.
There was a significant increase in the frequency of renal tubular cell karyomegaly and cytomegaly and renal adenoma in the animals treated with 1.0 or 2.5 mg/kg/day of auranofin and 2 or 6 mg/kg twice weekly of gold sodium thiomalate. Malignant renal epithelial tumors were seen in the 1.0 mg/kg/day and the 2.5 mg/kg/day auranofin and in the 6 mg/kg twice weekly gold sodium thiomalate-treated animals.
In a 12-month study, rats treated with auranofin at 23 mg/kg/day (192 times the human dose) developed tumors of the renal tubular epithelium, whereas those treated with 3.6 mg/ kg/day (30 times the human dose) did not.
In an 18-month study in mice given oral auranofin at doses of 1, 3 and 9 mg/kg/day (8, 24 and 72 times the human dose), there was no statistically significant increase above controls in the instances of tumors.
In the mouse lymphoma forward mutation assay, auranofin at high concentrations (313 to 700 ng/mL) induced increases in the mutation frequencies in the presence of a rat liver microsomal preparation.
Auranofin produced no mutation effects in the Ames test (Salmonella), in the *in vitro* assay (Forward and Reverse Mutation Inducement Assay with Saccharomyces), in the *in vitro* transformation of BALB/T3 cell mouse assay or in the Dominant Lethal Assay.
Pregnancy: Teratogenic Effects— Pregnancy Category C. Use of RIDAURA (auranofin) by pregnant women is not recommended. Furthermore, women of childbearing potential should be warned of the potential risks of RIDAURA therapy during pregnancy. (See below.)
Pregnant rabbits given auranofin at doses of 0.5, 3 or 6 mg/kg/day (4.2 to 50 times the human dose) had impaired food intake, decreased maternal weights, decreased fetal weights and an increase above controls in the incidence of resorptions, abortions and congenital abnormalities, mainly abdominal defects such as gastroschisis and umbilical hernia. Pregnant rats given auranofin at a dose of 5 mg/kg/day (42 times the human dose) had an increase above controls in the incidence of resorptions and a decrease in litter size and weight linked to maternal toxicity. No such effects were found in rats given 2.5 mg/kg/day (21 times the human dose).
Pregnant mice given auranofin at a dose of 5 mg/kg/day (42 times the human dose) had no teratogenic effects.
There are no adequate and well-controlled RIDAURA studies in pregnant women.
Nursing Mothers: Nursing during RIDAURA therapy is not recommended.
Following auranofin administration to rats and mice, gold is excreted in milk. Following the administration of injectable gold, gold appears in the milk of nursing women; human data on auranofin are not available.

Pediatric Use: RIDAURA (auranofin) is not recommended for use in pediatric patients because its safety and effectiveness have not been established.

ADVERSE REACTIONS
The adverse reactions incidences listed below are based on observations of 1) 4,784 RIDAURA treated patients in clinical trials (2,474 U.S., 2,310 foreign), of whom 2,729 were treated more than one year and 573 for more than three years; and 2) postmarketing experience. The highest incidence is during the first six months of treatment; however, reactions can occur after many months of therapy. With rare exceptions, all patients were on concomitant nonsteroidal anti-inflammatory therapy; some of them were also taking low dosages of corticosteroids.

Reactions occurring in more than 1% of RIDAURA-treated patients

Gastrointestinal: loose stools or diarrhea (47%); abdominal pain (14%); nausea with or without vomiting (10%); constipation; anorexia*; flatulence*; dyspepsia*; dysgeusia.

Dermatological: rash (24%); pruritus (17%); hair loss; urticaria.

Mucous Membrane: stomatitis (13%); conjunctivitis*; glossitis.

Hematological: anemia; leukopenia; thrombocytopenia; eosinophilia.

Renal: proteinuria*; hematuria.

Hepatic: elevated liver enzymes.
*Reactions marked with an asterisk occurred in 3-9% of the patients. The other reactions listed occurred in 1-3%.

Reactions occurring in less than 1% of RIDAURA-treated patients
Gastrointestinal: dysphagia; gastrointestinal bleeding†; melena†; positive stool for occult blood†; ulcerative enterocolitis.

Dermatological: angioedema.

Mucous Membrane: gingivitis†.

Hematological: aplastic anemia; neutropenia†; agranulocytosis; pure red cell aplasia; pancytopenia.

Hepatic: jaundice.

Respiratory: interstitial pneumonitis.

Neurological: peripheral neuropathy.

Ocular: gold deposits in the lens or cornea unassociated clinically with eye disorders or visual impairment.

† Reactions marked with a dagger occurred in 0.1-1% of the patients. The other reactions listed occurred in less than 0.1%.

Reactions reported with injectable gold preparations, but not with RIDAURA (auranofin) (based on clinical trials and on postmarketing experience)

Cutaneous Reactions: generalized exfoliative dermatitis.

Incidence of Adverse Reactions for Specific Categories—18 Comparative Trials

	RIDAURA (445 patients)	Injectable Gold (445 patients)
Proteinuria	0.9%	5.4%
Rash	26%	39%
Diarrhea	42.5%	13%
Stomatitis	13%	18%
Anemia	3.1%	2.7%
Leukopenia	1.3%	2.2%
Thrombocytopenia	0.9%	2.2%
Elevated liver function tests	1.9%	1.7%
Pulmonary	0.2%	0.2%

OVERDOSAGE

The acute oral LD50 for auranofin is 310 mg/kg in adult mice and 265 mg/ kg in adult rats. The minimum lethal dose in rats is 30 mg/kg.
In case of acute overdosage, immediate induction of emesis or gastric lavage and appropriate supportive therapy are recommended.
RIDAURA overdosage experience is limited. A 50-year-old female, previously on 6 mg RIDAURA daily, took 27 mg (9 capsules) daily for 10 days and developed an encephalopathy and peripheral neuropathy. RIDAURA was discontinued and she eventually recovered.
There has been no experience with treating RIDAURA overdosage with modalities such as chelating agents. However, they have been used with injectable gold and may be considered for RIDAURA overdosage.

DOSAGE AND ADMINISTRATION

Usual Adult Dosage: The usual adult dosage of RIDAURA (auranofin) is 6 mg daily, given either as 3 mg twice daily or 6 mg once daily. Initiation of therapy at dosages exceeding 6 mg daily is not recommended because it is associated with an increased incidence of diarrhea. If response is inadequate after six months, an increase to 9 mg (3 mg three times daily) may be tolerated. If response remains inadequate after a three-month trial of 9 mg daily, RIDAURA therapy should be discontinued. Safety at dosages exceeding 9 mg daily has not been studied.

Transferring from Injectable Gold:

In controlled clinical studies, patients on injectable gold have been transferred to RIDAURA (auranofin) by discontinuing the injectable agent and starting oral therapy with RIDAURA, 6 mg daily. When patients are transferred to RIDAURA, they should be informed of its adverse reaction profile, in particular the gastrointestinal reactions. (See PRECAUTIONS— Information for Patients.) At six months, control of disease activity of patients transferred to RIDAURA and those maintained on the injectable agent was not different. Data beyond six months are not available.

HOW SUPPLIED

Capsules, containing 3 mg auranofin, in bottles of 60.
NDC 65483-093-06

STORAGE AND HANDLING

Store between 15° and 30°C (59° and 86°F). Dispense in a tight, light-resistant container.

REVISED January 2011
©2007 Prometheus Laboratories Inc.
All rights reserved.

RIDAURA is a registered trademark of Prometheus Laboratories Inc.

Manufactured for:
Prometheus Laboratories Inc.
San Diego, CA 92121-4203

RI002E
