# Supplementary material for: In silico and in vitro drug screening identifies new therapeutic approaches for Ewing sarcoma
Source: Oncotarget. 2016 Nov 16;8(3):4079–95. doi: 10.18632/oncotarget.13385 (PMC5354814; doi:10.18632/oncotarget.13385)
Supplement: Supplementary file 3 [file oncotarget-08-4079-s003.pdf]

PRODUCT MONOGRAPH

RIDAURA®

Auranofin

Capsules 3 mg

ANTIRHEUMATIC AGENT

Xediton Pharmaceuticals Inc.
2000 Argentia Road,
Building 4, Suite 495
Mississauga, Ontario L5N 1W1

Date of Preparation:
January 28, 2010

Control No. 135899

PRODUCT MONOGRAPH

NAME OF DRUG

RIDAURA®

(Auranofin)

Capsules 3 mg

PHARMACOLOGICAL CLASSIFICATION

ANTIRHEUMATIC AGENT

ACTIONS AND CLINICAL PHARMACOLOGY

Ridaura® (auranofin) is a gold preparation and therefore has the potential for serious gold toxicity. The mechanism by which auranofin exerts its therapeutic effect has not been established.

In patients with adult rheumatoid arthritis or psoriatic arthritis, Ridaura® may modify disease activity as manifested by synovitis and associated symptoms, and reflected by laboratory parameters such as elevated ESR. There is no substantial evidence, however, that gold-containing compounds induce remission of rheumatoid arthritis.

Clinically the usual time of onset of therapeutic response to Ridaura® is 3 to 4 months. Continuing therapy beyond this time depends upon patient responsiveness, which includes improvement in parameters such as joint swelling, tenderness, pain, morning stiffness and grip strength. Continuing therapy beyond 6 months is unwarranted in patients showing insufficient improvement in the above parameters, and auranofin should be discontinued because of potential serious adverse reactions.

PHARMACOKINETICS

In 5 rheumatoid arthritic patients, the oral administration of a single 6 mg (equivalent to 1.74 mg of gold) dose of a solution of radiolabelled auranofin demonstrated that approximately 25% of the oral dose was absorbed. Peak plasma radioactive gold concentrations of 0.039 to 0.11 pg ^{195}Au/mL were reached in 1.5 to 2.5 hours. The mean plasma terminal half-life was 17 days, while the mean total body terminal half-life was 58 days.. By day 10 post-administration, 77% of the initially administered labelled gold had been excreted, 73% in the feces and 4% in the urine. Six months after this single dose, approximately 99.6% of the initially administered labelled gold had been excreted, with 0.4% retained in the body.

Following 6 months of therapy with unlabelled auranofin 3 mg BID a single 6 mg dose of radiolabelled auranofin was administered. Peak plasma radioactive gold concentrations of 0.027 to 0.138 pg ^{195}Au/mL were reached in 1.0 to 1.5 hours. The mean plasma half-life was 25.5 days, while the mean total body terminal half-life was 80.8 days. By day 10 post-administration, 75% of the labelled gold had been excreted, 70% in feces and 5% in the urine.

In clinical studies, steady state blood gold levels were achieved in about 3 months. With auranofin at 6 mg/day, mean blood gold levels of 0.62 ± 0.195 pg/mL (91 patients) have been observed after 3 months of treatment and 0.68 ± 0.452 pg/mL (63 patients) after 6 months. In blood, approximately 40% of auranofin gold is associated with red cells and 60 % associated with serum proteins. In contrast, 99% of injectable gold is associated with serum proteins.

Mean blood-gold concentrations are proportional to dose; however, no correlation between blood-gold concentrations and safety or efficacy has been established.

INDICATIONS AND CLINICAL USE

Ridaura® (auranofin) is indicated in the management of adults with active (classical or definite) rheumatoid arthritis who have not responded to adequate trials of conventional anti-inflammatory therapy. Ridaura® might also be of benefit in patients with psoriatic arthritis.

Ridaura® should be considered only when salicylates or other non-steroidal anti-inflammatory drugs, and, when appropriate, steroids, have proven to be inadequate for controlling the symptoms of rheumatoid arthritis.

Physicians planning to use Ridaura® should be experienced with chrysotherapy and should thoroughly familiarize themselves with the toxicity and benefits of Ridaura®.

In controlled clinical trials, comparing Ridaura® with injectable gold, Ridaura® was associated with fewer drop-outs due to adverse reactions, while injectable gold was associated with fewer drop-outs for inadequate or poor therapeutic effects. Physicians should consider these findings when deciding on the use of Ridaura® in patients who are candidates for chrysotherapy.

Ridaura® should be added to an ongoing comprehensive treatment program which includes physical as well as other drug therapy. The usual time to onset of therapeutic response to Ridaura® is 3 to 4 months; some patients require as long as 6 months to show a full clinical response.

Ridaura® is not indicated in other arthropathies, such as osteoarthritis.

CONTRAINDICATIONS

Ridaura® (auranofin) is contraindicated in patients with a history of serious gold-induced toxicity including necrotizing enterocolitis, pulmonary fibrosis, exfoliative dermatitis or hypersensitivity. Ridaura® should not be prescribed for patients with progressive renal disease, severe hepatological disorders.

Use in Pregnancy:

Ridaura® has been shown to be embryotoxic in rats at dose levels of 5 mg/kg/day or higher and both embryotoxic and teratogenic in rabbits at doses of 0.5 mg/kg/day or higher. Therefore, Ridaura® should not be given to pregnant women. Furthermore, women of childbearing potential should be made aware of the necessity to avoid pregnancy during treatment and for at least six months after because of the slow excretion of gold and its persistence in the body tissues after discontinuation of treatment.

Nursing Mothers:

Gold is excreted in rodent milk following the administration of auranofin. It is not known whether Ridaura® is excreted in human milk; however, injectable gold appears in the milk of nursing mothers following administration. Therefore, it is recommended that Ridaura® not be given during nursing.

WARNINGS

Ridaura® (auranofin) contains gold and, like other gold-containing drugs, can cause gold toxicity. Danger signs of possible gold toxicity include the following: fall in hemoglobin, leukopenia below 4000 WBC/mm^3, granulocytes below 1500/mm^3, decrease in platelets below 150,000/mm^3, proteinuria, hematuria, pruritus, rash, stomatitis or persistent diarrhea. Therefore, it is recommended that white blood cells with differential, platelet count, haemoglobin, urinary protein and renal and liver function be measured prior to Ridaura® therapy to establish a baseline and to identify pre-existing conditions. See CONTRAINDICATIONS.

The possibility of adverse reactions should be explained to patients before staring therapy. See INFORMATION FOR PATIENTS.

Patients should be advised to report promptly any unusual signs and symptoms occurring during treatment with Ridaura®, such as sore throat or tongue, mouth ulceration, skin rash, easy bruising, purpura, epistaxis, bleeding gums or menorrhagia.

When the following adverse reactions occur, Ridaura® therapy may require modification or additional monitoring as outlined below:

Thrombocytopenia:

Thrombocytopenia has occurred in approximately 1 - 3% of patients (see ADVERSE REACTIONS) treated with Ridaura®, some of whom developed bleeding. The thrombocytopenia appears to be peripheral in origin in most cases and is usually reversible upon withdrawal of Ridaura®. Its onset bears no relationship to the duration of Ridaura® therapy and its course may be rapid. While patients' platelet counts require monitoring

every 2 weeks for the first 3 months and at least monthly thereafter, the occurrence of a precipitous decline in platelets or a platelet count less than 100,000/mm^3 or signs and symptoms (e.g., purpura, ecchymoses or petechiae) suggestive of thrombocytopenia indicates a need to immediately withdraw Ridaura® and all other therapies with the potential to cause thrombocytopenia, and to obtain additional platelet counts. No additional Ridaura® should be given unless the thrombocytopenia resolves and further studies show it was not due to gold therapy.

Proteinuria:

Proteinuria has developed in approximately 3 - 9% of patients (see ADVERSE REACTIONS) treated with Ridaura®. Urinalysis should be performed every 2 weeks for the first 3 months and at least monthly thereafter. If clinically significant proteinuria or microscopic hematuria is found, Ridaura® and all other therapies with the potential to cause proteinuria or microscopic hematuria should be stopped immediately.

There has been no experience with the concomitant use of Ridaura® and penicillamine, chloroquine/hydroxychloroquine, immunosuppressive agents (e.g., cyclophosphamide, azathioprine, or methotrexate) or high doses of corticosteroids and therefore, such use cannot be recommended.

PRECAUTIONS

General:

The potential benefits of using Ridaura® (auranofin) in patients with inflammatory bowel disease, skin rash or history of bone marrow depression, should be weighed against: 1) the potential risks of gold toxicity on organ systems previously compromised or with decreased reserve, and, 2) the difficulty in quickly detecting and correctly attributing the toxic effect.

The following adverse reactions have been reported with the use of gold preparations and require modification of Ridaura® treatment or additional monitoring. See ADVERSE REACTIONS for the approximate incidence of those reactions specifically reported with Ridaura®.

Gastrointestinal Reactions:

Gastrointestinal reactions reported with gold therapy include diarrhea/loose stools, nausea, vomiting, anorexia and abdominal cramps. The most common reaction to Ridaura® is diarrhea/loose stools reported in approximately 47% of patients. This is generally manageable by reducing the dosage (e.g., from 6 mg daily to 3 mg). In 4% of the patients it has been necessary to discontinue Ridaura® permanently.

Ulcerative enterocolitis is a rare serious gold reaction. Therefore, patients with gastrointestinal symptoms should be monitored for the appearance of gastrointestinal bleeding.

Cutaneous Reactions:

Dermatitis is the most common reaction to parenteral gold therapy and the second most common reaction to Ridaura®. Any eruption especially if pruritic that develops during treatment should be considered a gold reaction until proven otherwise. Pruritus often exists before dermatitis becomes apparent, and therefore should be considered to be a warning signal of a cutaneous reaction. Gold dermatitis may be aggravated by exposure to sunlight; an actinic rash may develop. The most serious form of cutaneous reaction reported with parenteral gold is generalized exfoliative dermatitis.

In patients with psoriatic arthritis who were involved in all clinical trials with Ridaura®, 5/438 (1.1%) Ridaura®-treated patients and 2/183 (1.1%) placebo-treated patients had an exacerbation of their psoriasis requiring withdrawal.

Mucous Membrane Reactions:

Stomatitis, another common gold reaction, may be manifested by shallow ulcers on the buccal membranes, on the borders of the tongue, and on the palate or in the pharynx. Stomatitis may occur as the only adverse reaction or with a dermatitis. Sometimes diffuse glossitis or gingivitis develops. A metallic taste may precede these oral mucous membrane reactions and should be considered a warning signal.

Renal Reactions:

Ridaura®, as other gold preparations, can produce a nephrotic syndrome or glomerulitis with proteinuria and hematuria. These renal reactions are usually relatively mild and subside completely if recognized early and treatment is discontinued. They may become severe and chronic if treatment is continued after the onset of the reaction. Therefore, it is important to perform urinalysis regularly and to discontinue treatment promptly if proteinuria or hematuria develops.

Hematologic Reactions:

Blood dyscrasias including leukopenia, granulocytopenia and thrombocytopenia have all been reported as reactions to injectable gold and Ridaura®. These reactions may occur separately or in combination at any time during treatment. In addition, a case of pure red cell aplasia has been reported as a reaction Ridaura®. Because these reactions have potentially serious consequences, blood dyscrasias should be constantly watched for through monitoring of the formed elements of the blood, every 2 weeks for the first 3 months and at least monthly thereafter.

Ocular:

There have been some reports of gold deposits in the lens or corneas of patients treated with Ridaura®. These deposits have not led to any eye disorders or any degree of visual impairment, and have cleared within 3-6 months of cessation of therapy. Initial and periodic ophthalmic examinations are recommended in patients being treated with auranofin.

Miscellaneous Reactions:,

Rare reactions attributed to gold include cholestatic jaundice; gold bronchitis and interstitial pneumonitis and fibrosis; peripheral neuropthy; partial or complete hair loss; fever. The physician should be constantly on guard for any of the above changes, and as a precaution, suitable laboratory monitoring should be done at appropriate intervals.

Children:

The safety and effectiveness of Ridaura® in children under age 16 have not yet been established. Consequently, use in this age group cannot be recommended.

Carcinogenesis:

In a 24-month study in rats, animals treated with auranofin at 0.4, 1.0 or 2.5 mg/kg/day orally (3, 8 or 21 times the human dose) or gold sodium thiomalate at 2 or 6 mg/kg injected twice weekly (4 or 12 times the human dose) were compared to untreated control animals. There was a significant increase in instances of renal tubular cell karyomegaly and cytomegaly and renal adenoma in the animals treated with 1.0 or 2.5 mg/kg/day of auranofin and 2 or 6 mg/kg twice weekly of gold sodium thiomalate. Malignant renal epithelial tumors were seen in the 2.5 mg/kg/day auranofin and in the 6 mg/kg twice weekly gold sodium thiomalate-treated animals.

In a 12-month study, rats treated with auranofin at 23 mg/kg/day (192 times the human dose) developed adenomas of the renal tubular epithelium, whereas those treated with 3.6 mg/kg/day (30 times the human dose) did not.

Drug Interactions:

One report suggests that, in a single patient, concurrent administration of Ridaura® and phenytoin was associated with increased phenytoin blood levels.

ADVERSE REACTIONS

The adverse reactions listed below are based on observations on 4784 rheumatoid arthritis patients treated with Ridaura® (auranofin) of whom 2729 were treated for more than 1 year and 573 for more than 3 years. The overall incidence of adverse reactions was 62%, of whom 18.6% discontinued therapy. The most common adverse reactions were diarrhea (47%), rash (24%) pruritus (17%), abdominal pain (14%) and stomatitis (13%). More serious adverse reactions were anemia (1.6%), leukopenia (1.9%), thrombocytopenia (0.9%) and proteinuria (5.0%). The highest incidence was during the first 6 months of treatment. However, reactions can occur at any time throughout the course of therapy.

Clinical trials were conducted assessing Ridaura® in the treatment of 438 psoriatic arthritis patients. The nature and incidence of adverse reactions were similar to those observed in rheumatoid arthritis patients.

Reactions occurring in more than 1% of Ridaura®-treated patients

Gastrointestinal:
Loose stools or diarrhea (47%); abdominal pain (14%); nausea with or without vomiting (10%); anorexia*; flatulence*; constipation and dysgeusia
Dermatological:
Rash (24%); pruritus (17%); hair loss; urticaria
Mucous membrane:
Stomatitis (13%); conjunctivitis*; glossitis
Hematological:
Anemia; leukopenia; thrombocytopenia; eosinophilia
Renal:
Proteinuria*; hematuria
Hepatic:
Elevated liver enzymes
Miscellaneous:
Weight loss

Reactions occurring in less than 1% of Ridaura-treated patients

Gastrointestinal:
Gastrointestinal bleeding; melena; positive stool for occult blood; dysphagia (<0.1%); ulcerative enterocolitis (<0.1%)
Dermatological:
Angioedema (<0.1%)
Mucous membrane:
Gingivitis
Hematological:
Neutropenia; agranulocytosis (<0.1%), aplastic anemia (<0.1%)
Renal:
Membranous glomerulonephritis (<0.1%), nephrotic syndrome (<0.1%)

Hepatic:
Jaundice (<0.1 %)

Respiratory:
Interstitial pneumonitis (<0.1%)
Neurological:
Peripheral neuropathy (<0.1%)

(* Reactions marked with an asterisk occurred in 3-9% of the patients. The other reactions listed occurred in 1-3%)

SYMPTOMS AND TREATMENT OF OVERDOSAGE

In case of acute overdosage, immediate induction of emesis or gastric lavage and appropriate supportive therapy are recommended.

Ridaura® overdosage experience is limited. A 50-year-old female, previously on 6 mg Ridaura® daily, took 27 mg (9 capsules) daily for 10 days and developed an encephalopathy and peripheral neuropathy. Ridaura® was discontinued and she eventually recovered.

There has been no experience with treating Ridaura® overdosage with modalities such as chelating agents; however, they have been used with injectable gold and may be considered when treating Ridaura® overdosage.

DOSAGE AND ADMINISTRATION

The usual adult starting dosage is 6 mg per day. This dose may be given:

twice a day: one 3 mg capsule with breakfast and one with the evening meal
OR
once a day: two 3 mg capsules with breakfast
OR
two 3 mg capsules with the evening meal

Ridaura® (auranofin) should be discontinued in those patients in whom no response is observed after 4 months administration. In those patients in whom a partial response is observed after 4 months, Ridaura® may be continued at 6 mg/day, or the dose may be increased to 9 mg/day (one 3 mg capsule 3 times a day), for an additional 2 months. Ridaura® should be discontinued in patients in whom a satisfactory clinical response has not occurred after 6 months treatment. Daily dosages above 9 mg are not recommended.

Because of possible serious adverse reactions, some rheumatologists suggest reducing the dosage or discontinuing gold altogether when patients are in clinical remission (ARA criteria) lasting for at least six months, keeping in mind that cessation of therapy may allow the disease to progress further. Each patient must be evaluated individually.

Transferring from Injectable Gold:

In a controlled clinical trial, patients on injectable gold were transferred to Ridaura® by discontinuing the injectable agent and starting oral therapy with Ridaura®, 6 mg daily. At six months, control of disease activity of patients transferred to Ridaura® and those maintained on the injectable agent was not different. Data beyond six months are not available. When patients are transferred to Ridaura®, they should be informed of its adverse reaction profile, in particular the gastrointestinal reactions. See PRECAUTIONS.

PHARMACEUTICAL INFORMATION

Chemical Name: Auranofin is 2,3,4,6-tetra-0-acetyl-1-thio-(3-D-glucopyranosato-S-(triethylphosphine) gold.

Structural Formula:

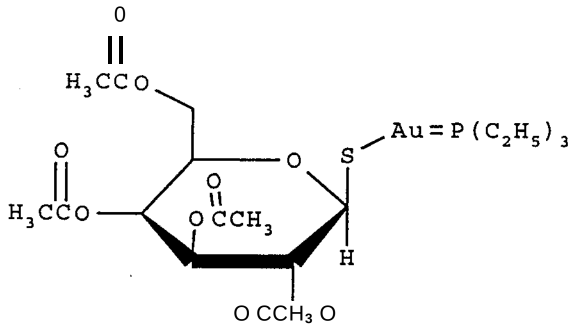

Molecular Formula: C20H34Au09SP Molecular Weight: 678.49

Gold Content: 29%

Description: A white odorless crystalline powder.

Storage Recommendations

Store at room temperature (15 - 30 C°). Dispense in a tight, light resistant container.

AVAILABILITY OF DOSAGE FORMS

Capsules:

Each Ridaura® package contains a bottle of 60 tan and brown opaque Ridaura® capsules 3 mg, monogrammed Ridaura, and a package insert packed in an outer carton.

INFORMATION FOR THE CONSUMER

Educational information for patients beginning Ridaura® therapy is available from the manufacturer upon request. The information provided is as follows:

Understanding Rheumatoid Arthritis and Psoriatic Arthritis

Your doctor has prescribed Ridaura® (oral gold) as part of your treatment program. Because you and your family may wish to know more about the disease and its treatment, Pharmascience has developed this informative booklet and a companion cassette tape to supplement the information your doctor has given you. These are provided at no additional charge with your first month's supply of Ridaura® capsules.

Your doctor has decided that Ridaura® is a proper medication for you; it may not be appropriate for others. Do not give any Ridaura® capsules to other family members or friends who may appear to have a condition similar to yours. Advise them to consult their doctor for proper diagnosis and treatment.

Rheumatoid arthritis is a disease affecting primarily the joints, causing joint swelling, pain and stiffness.

It is a fairly common ailment, affecting about 1% of the population. The disease occurs most frequently in adults (ages 20 to 60), but it may develop at any age. Rheumatoid arthritis is three times more common in women than in men.

Psoriatic arthritis is a disease affecting primarily the joints, causing joint swelling, pain and stiffness and is also associated with skin or nail lesions of psoriasis.

In the general populations, 1% - 2% are affected by psoriatic arthritis. The disease occurs most frequently in adults ages 30 - 50 years. Psoriasis precedes the onset of arthritis in approximately 75% of the patients, and occurs simultaneously in about 15%. In a small number of patients, arthritis precedes the appearance of skin lesions.

In order to understand the nature of the disease, it is helpful to review the joint's normal structure.

The Normal Joint

A joint is an area where two bones meet end-to-end. Joints can be thought of as hinges that enable us to walk, lift objects, bend, turn, and sit down. Muscles and ligaments support the joint, and keep the bones aligned within the joint capsule, permitting movement and providing protection from injury. The ends of the opposing bones are covered by tough elastic sheaths of cartilage and separated by a pocket of fluid - synovial fluid - which is produced by the synovium (the inner lining of the joint capsule). Synovial fluid has two main functions: 1) to lubricate the cartilage, reducing the mechanical stress caused by movement, and 2) to supply nutrients that maintain the health of surrounding tissues. Disorders such as rheumatoid arthritis that adversely affect the synovium or synovial fluid will alter the joint's delicate architecture, possibly impairing joint function and movement.

The Arthritic Joint

The causes of rheumatoid and psoriatic arthritis are unknown.

Whatever the cause, the result is joint inflammation and its accompanying symptoms and, later, joint damage. Inflammation attracts blood components that release enzymes capable of attacking the synovium. Once irritated and inflamed, the synovium produces excess fluid, which puts painful pressure on surrounding tissues. Cellular debris from the inflammatory process, called pannus, accumulates on the cartilage surfaces and releases enzymes that can destroy the joint tissues and, eventually, the bone. Such damage is irreversible, eventually limiting joint motion.

Disease Symptoms and Diagnosis

The first symptom of rheumatoid arthritis is usually general fatigue, accompanied by overall muscle soreness, stiffness, aches and pain. Joint inflammation is marked by pain, swelling, warmth and tenderness of one or more joints in the hands or feet. It can also affect the wrist, shoulders, elbows, hips and knees.

Once rheumatoid arthritis develops, it may progress over many months and years. Symptoms and discomfort can vary greatly from day to day or month to month. There may be repeated and prolonged periods when symptoms disappear and discomfort is greatly reduced (apparent remissions), as well as episodes when symptoms intensify (exacerbations), increasing discomfort.

A diagnosis of rheumatoid arthritis is based on the patient's history of symptoms and an examination of joint involvement. The results of x-rays and blood tests for inflammation and rheumatoid arthritis markets may also contribute to the diagnosis. A diagnosis of psoriatic arthritis is based on the presence of symptoms of inflammatory arthritis coupled with typical skin or nail lesions of psoriasis.

Rheumatoid and Psoriatic Arthritis Treatment Programs

Although there is presently no known cure for rheumatoid arthritis or psoriatic arthritis, much can be done to relieve pain, improve function and, in some cases, delay further joint damage. Given the unpredictable nature of rheumatoid arthritis, the best treatment usually requires several therapeutic measures in a total management program. Such programs include rest, limited physical exercise and medication.

Lifestyle

Your doctor may recommend altering your daily routine to include periods of rest and exercise. With the support of physician, family and friends, this will help you feel better and maintain an active lifestyle.

Because rheumatoid arthritis causes fatigue, adequate rest is an integral part of therapy. Your doctor will probably recommend that you take short rest periods during the day and get a full night's sleep. Bed rest is usually required only during severe flare-ups. Too much rest can actually make you stiffer and weaker.

It is important not to overwork any part of your body that is affected by the disease. In some cases, it may be necessary to change your routine at home or work to prevent excessive fatigue.

Your doctor may recommend specific exercises. These regular but limited exercises are intended to prevent joint stiffness and reduced motion. The doctor may also recommend whirlpool baths, the use of supports or splints, or the application of warm or cold compresses.

A healthy lifestyle should also include a well-balanced diet (not fad diets).
Although these measures can be effective, they are usually accompanied by drug therapy used to relieve symptoms. In some severe cases, programs incorporate certain drugs that can influence the course of the disease.

Drug Therapy

In most psoriatic arthritis patients, psoriasis is successfully treated with creams or ointments; in some patients, the psoriasis requires no therapy at all. Adequate control of skin disease may lead to improvement of the joint disease in an occasional patient.

Some patients with psoriatic arthritis will have more progressive joint disease and should be treated similarly to those with progressive rheumatoid arthritis.

Because rheumatoid arthritis and psoriatic arthritis are chronic diseases, drug therapy is long-term. The goals of drug therapy are to relieve pain and, if possible, slow the progression of the disease. Drugs used in the management of rheumatoid arthritis and mild to moderate psoriatic arthritis can be divided into two groups.

First, there are drugs such as acetylsalicylic acid (ASA, e.g., Aspirin®) and other nonsteroidal anti-inflammatory drugs (NSAIDs) that combat inflammation and provide relief of pain and other symptoms of rheumatoid arthritis. You have been taking one of these drugs. However, because of insufficient response to this drug and the nature of your disease, your doctor has decided that other drug measures, which may modify the progress of the disease, may be beneficial.

These other drugs are intended to slow the progress of rheumatoid arthritis. Such drugs include injectable gold, Ridaura®, penicillamine, and certain drugs that suppress the immune system. Although effective, these drugs do not provide immediate relief from pain or stiffness, and are normally given with ASA or other NSAIDs.

All these drugs have some potentially serious side-effects and you must keep your appointments with your doctor who will monitor your condition carefully.

Facts about Ridaura®

As part of your overall treatment program, your doctor has prescribed Ridaura® - oral gold. The recommended dose for initial therapy is two capsules per day.

Clinical experience has demonstrated that Ridaura® is effective in the long-term management of rheumatoid and psoriatic arthritis - relieving pain, morning stiffness and fatigue, reducing the number of tender or swollen joints, and improving daily activity. Ridaura® may also modify the progression of the disease. However, the precise mechanism of action of Ridaura® is still unknown.

The beneficial effects of Ridaura® take time to become apparent. A response may be seen after 3 to 4 months, but it may take up to 6 months. Your doctor will instruct you to continue taking ASA or NSAIDs to relieve more immediate symptoms of rheumatoid arthritis.

Because your disease is chronic and took many months or even years to develop, it is important that you do not become discouraged if you do not feel significant improvement immediately with this regimen. Patience is the key. You should continue taking Ridaura® and other medications as your doctor instructs, even if you have no pain. Your doctor will be monitoring therapy with frequent laboratory tests and office visits and will make any necessary adjustments in your therapy.

Side effects may occur with Ridaura®. The most frequent adverse reaction to Ridaura® has been a change in stool pattern - loose stools or diarrhea. Stomach pain, nausea, skin rashes, itching, eye inflammation (conjunctivitis), and inflammation of the tissues of the mouth (stomatitis) have also been reported during therapy with Ridaura®. These events generally occur early in the treatment program and can usually be relieved without discontinuing Ridaura® therapy but sometimes discontinuation is necessary.

Rarely, more severe reactions occur which include changes in blood cells or kidneys. These can be reversed if discovered early. If you notice any unusual or troublesome symptoms including fever, sore throat, lesions in the mouth, spontaneous bruising, black or tarry stools, skin reactions, or persistent or severe indigestion while taking Ridaura® contact your doctor immediately.

In Summary

Millions of people are coping with rheumatoid arthritis and psoriatic arthritis. And like you, they have had to modify their lifestyle by changing their daily routines.

The treatment program that your doctor has recommended is designed to keep you active and feeling good. Pay careful attention to your doctor's instructions regarding regular rest periods, specific exercises, and medications.

BE SURE TO TAKE RIDAURA AND OTHER MEDICATIONS EXACTLY AS DIRECTED AND CONTACT YOUR DOCTOR IF YOU NOTICE ANY UNUSUAL OR TROUBLESOME SYMPTOMS.

Finally, if you have any questions concerning rheumatoid arthritis or Ridaura®, note these on the following pages and discuss them with your doctor during your next appointment. You may also wish to fill out the diary to follow your treatment instructions.

An abbreviated information leaflet accompanies all prescriptions. See AVAILABILITY. The text follows:

IMPORTANT

Information for the Patient

Your doctor has prescribed Ridaura® (oral gold) as part of your treatment program for arthritis.

Ridaura® is an oral preparation containing gold. Gold in the form of an injection has been used in treatment of severe rheumatoid arthritis for a number of years. Clinical experience has shown that Ridaura® is helpful in the management of rheumatoid arthritis which is not satisfactorily controlled by such drugs as acetylsalicylic acid (ASA, e.g., Aspirin®) or other non-steroidal anti-inflammatory drugs. Be sure to take Ridaura® and other medications exactly as directed. Do NOT increase dosage unless instructed to do so by your doctor.

Side effects may occur with Ridaura®. The most frequent adverse reaction to Ridaura® has been a change in stool pattern - loose stools or diarrhea. Stomach pain, nausea, skin rashes, itching, eye inflammation (conjunctivitis), and inflammation of the tissues of the mouth (stomatitis) have also been reported during therapy with Ridaura®. These events generally occur early in the treatment program and can usually be relieved without discontinuing Ridaura® therapy but sometimes discontinuation is necessary.

Rarely, more severe reactions occur which include changes in blood cells or kidneys. These can be reversed if discovered early. Therefore, your doctor will be monitoring therapy with frequent laboratory tests and office visits and will make any necessary adjustments in your therapy. If you notice any unusual or troublesome symptoms including fever, sore throat, lesions in the mouth, spontaneous bruising, black or tarry stools, skin reactions, or persistent or severe indigestion while taking Ridaura®, contact your doctor immediately.

Although your doctor has decided that Ridaura® is a proper medication for you, it may not be appropriate for others. Do NOT give any Ridaura® capsules to family members or friends who may appear to have a condition similar to yours. Advise them to consult their doctor for proper diagnosis and treatment.

The treatment program that your doctor has recommended is designed to keep you active and feeling good. Pay careful attention to your doctor's instructions regarding regular rest periods, specific exercises, and medications.

PHARMACOLOGY

Immunology

Auranofin affects cell-mediated immunity in vivo and in vitro. In the mouse, auranofin (0.625 - 10 mg gold/kg p.o.) caused a stimulatory effect on the suboptimal delayed hypersensitivity response induced by sheep red blood cells (SRBC); auranofin (5 and 10 mg gold/kg p.o.) also stimulated low-grade oxazolone-induced contact sensitivity.

In in vitro experiments auranofin (0.1 - 1.0 µg auranofin/mL) inhibited human lymphocyte phytohemagglutinin-induced mitogenesis, human neutrophil and monocyte chemotaxis, human monocyte phagocytosis, and human polymorphonuclear neutrophil superoxide production and phagocytosis. The activity of natural killer cells was affected by auranofin in vitro in a biphasic manner, being stimulated at low concentrations (0.125 - 0.5 µg auranofin/mL) and inhibited at high concentrations (1.0 - 2.5 µg auranofin/mL).

In vitro, auranofin significantly reduced particle-stimulated lysosomal enzyme release from rate leukocytes over a wide range of concentrations (10^{-3} to 10^{-6}M). Since enzyme activity per se was not inhibited, the drug-induced decrease in lysosomal enzyme levels was apparently due to inhibition of extracellular release.

Auranofin has been shown to inhibit platelet aggregation. Using platelet-rich plasma obtained from human blood, auranofin, at a concentration of 10 µg/mL, was a potent inhibitor of epinephrine-, ADP-, or collagen-induced platelet aggregation.

The effect auranofin on antibody-dependent cell-mediated cytotoxicity (ADCC) was determined by measuring ^{51}Cr released from mouse L929 fibroblasts (which had been incubated with antibody and splenic lymphocytes or PMN leukocytes, from adjuvant-arthritic rats). ADCC was inhibited at 1µM auranofin. Auranofin also reduced the ADCC of human monocytes (0.125 µg auranofin/mL) and lymphocytes (1.25 µg auranofin/mL).

Auranofin has been shown to affect humoral immunity in vivo. Auranofin administration (5 and 10 µg gold/kg for 2 weeks) to adjuvant-arthritic rats suppressed hemagglutinating IgG antibodies to SRBC, and antibodies to mouse fibroblasts. Measurements of murine antibody production against SRBC, using hemolytic plaque-form cells, showed inconsistent inhibition. The effect of auranofin on humoral immunity has also been investigated in in vitro studies.

Pre-treatment of mouse spleen cells with auranofin (1 x 10^{-4}M and 1 x 10^{-5}M) resulted in a marked inhibition of plaque-forming cells. Auranofin (2.5 µg/mL) also inhibited pokeweed mitogen-induced immunoglobulin production.

The effect of auranofin on immediate-type hypersensitivity was illustrated in four models: inhibition of rat 48-hour passive cutaneous anaphylaxis after intravenous dosing (20 mg auranofin/kg); inhibition of anti-human IgE-induced release of histamine and SRS-A from Rhesus monkey lung (1.5 x 10^{-4}M); inhibition of histamine release from passively sensitized fragmented Rhesus monkey skin (1.5 x 10^{-4}M); and inhibition of ovalbumin antigen-induced release of histamine from passively sensitized, fragmented rat lung (1.5 x 10^{-4}M).

Pharmacokinetics

In animals, auranofin absorption varied from 19% (rat) to 26% (dog) after oral dosing. Peak concentrations of gold in blood occurred 6.5 hours after dosing in dogs. Auranofin gold in the blood was found mainly in the red blood cells and plasma proteins (rat, 65% and 35%; dog, 82% and 17%; respectively).

The mean blood half-life of gold derived from auranofin was 1.6 days in the rat and 2.7 days in the dog. In rat tissues, gold concentration was highest in the kidneys (cortex more than medulla), followed by gut, liver, spleen and lungs.

The principal route of elimination of auranofin is the feces: 79% of a single dose within 21 days after oral administration in the rat; 83% within 21 days in the dog. Corresponding urinary excretion was 10% and 16%.

Gold derived from auranofin crosses the placental barrier and is secreted in the milk of lactating rats.

Studies with radiolabelled auranofin indicated that the auranofin molecule fragments into three fractions: a protein-gold complex, triethylphosphine oxide, and acetyl thioglucose. Triethylphosphine oxide has been isolated from rat and dog urine and identified; it has also been isolated in the urine of rheumatoid arthritis patients receiving auranofin.

Pharmacodynamics

In animal studies auranofin had no significant effect on hemodynamic and electrocardiographic parameters, pulmonary function, central nervous system activity, gastric mucosa or the endocrine system. Auranofin showed a slight antidiuretic effect in rats (2 - 40 mg auranofin/kg p.o.) but not in dogs (0.6 - 1.2 mg auranofin/kg p.o.). Auranofin (up to 15 mg gold/kg p.o.) failed to induce liver enzyme activity in rats.

TOXICOLOGY

Acute Toxicity Studies

Animal Species	Sex	Doses Used (mg/kg)	Route	LD_{50} (mg/kg)
Mouse CF1	M	150-1000	PO	310
	F	90-1000	PO	225
Mouse B6C3F1	M	100-600	PO	190
	F	65-600	PO	187
Rat CD	M	50-525	PO	265
	F	50-525	PO	330

Signs of toxicity occurred as early as 15-20 minutes after dosing and included a marked decrease in spontaneous motor activity, low body posture, marked ptosis and lacrimation. The highest doses produced marked hypotonia, prostration and asphyxial convulsions.

Compounds which significantly potentiated auranofin's lethality

Auranofin's lethality (LD50) in rats was potentiated when given concomitantly with the usual human daily dose of warfarin; it was potentiated when given with five times and usual human daily dose of clonidine and propoxyphene; it was potentiated when given with ten times the usual human daily dose of acetaminophen, allopurinol, acetylsalicylic acid, chlorpropamide, furosemide, quinidine, sulindac and thioridazine; it was potentiated when given with twenty times the usual human dose of diphenoxylate, phenylpropanolamine and tolmetin.

Subacute Toxicity Studies

In rats, oral doses of 3.6, 12 and 36 mg auranofin/kg/day, (15/sex/dose) were administered for 3 months. Animals in the middle and high dose groups showed initial diarrhea (which subsided with continued dosing), initial increase in WBC count in high-dose females, and slight decrease in alkaline phosphatase. Dose-related increase in salivation, decrease in hemoglobin and hematocrit, and depression of the weight gain curve were also observed. In dogs, oral doses of 1.8, 3.6-7.2 and 6.9-18.0 mg auranofin/kg/day (3/sex/dose) were administered for 3 months. Doses were increased in the middle and high dose groups as much as the emesis and diarrhea would allow. Dose-related emesis, diarrhea, decreased food consumption and decreased weight gain were observed. In the high dose group, emaciation of the hind limbs was observed. There were dose-related decreases in hematocrit and hemoglobin values but no apparent change in erythrocyte morphology. Minimal reticulocytosis and erythroid hyperplasia occurred at the high dose.

Chronic Toxicity Studies

Auranofin was administered orally to rats at 3.6, 12 and 23 mg/kg/day (40/sex/dose) for 12 months. Dose-related salivation occurred throughout the study. soft stools occurred in the high dose group during the first week, and toward the end of the study. Increased muscle tone and excessive urination occurred in the high dose group. Drug-treated animals had lower weight gains than controls. Slight dose-related anemia and leukocytosis were observed. Histological examination revealed dose-related karyomegaly and cytomegaly of the renal tubular epithelial cells. Adenomas of the renal cortical tubular epithelium occurred in 2 females in the high dose group. There were dose-related incidences of ulcers at the ileocecocolic junction (usually associated with enlargement of mesenteric lymph nodes) and superficial erosion of the gastric mucosa.

Auranofin was administered orally to dogs at 0.6, 1.2 and 2.4-6.0 mg/kg/day (4/sex/dose) for 12 months. The dose was increased in the high dose group as much as the emesis and diarrhea would allow. Dose-related emesis occurred, the number of dogs affected decreasing with continued dosing. The incidence of diarrhea was dose-related; soft stools occurred sporadically. Salivation occurred in the high dose group (initially in the second month) and gradually involved all of the high dose dogs. Spontaneous motor activity decreased in high dose dogs toward the end of the study. The body weight of one high dose female was lower than normal during the last 4 months of the study. A dose-related subclinical anemia occurred

in the middle and high dose groups. Characteristics of the anemia (increased reticulocyte count, increased erythrocyte mean-cell volume, increased urine coproporphyrin, erythroid hyperplasia of the bone marrow, iron in renal tubular epithelial cells) showed it to be a chronic intravascular hemolytic anemia. Decreases in serum total protein, albumin, calcium, SGPT, and carbon dioxide content were dose-related. There was dose-related erythroid hyperplasia of the bone marrow, and hyperplasia of the follicular cells of the thyroid gland. There were no clinical changes indicating abnormal thyroid function.

Carcinogenicity Studies

Auranofin was administered orally to mice at doses of 1, 3 and 6-9 mg/kg/day (110/sex/dose) for 18 months. The high dose was raised from 6 to 9 mg/kg on day 294. This was done because of the low toxicity of the 6 mg/kg/day dose. Excessive salivation occurred initially in the fifteenth month; incidence was low and dose-related. There was an increase in benign hematomas among high dose males.

In a 24-month carcinogenicity study, rats were dosed orally at 0.4, 1.0 and 2.5 mg auranofin/kg/day (75/sex/dose). Salivation occurred, principally in the high dose group; it was first observed in the fourth month. A slightly lower mean body weight was seen in high dose males. There was a significant increase in the frequency of renal tubular cell karyomegaly and cytomegaly and renal adenoma in the animals treated with 1 or 2.5 mg auranofin/kg/day. Malignant renal epithelial tumours were seen in the high dose group.

Mutagenicity Studies

In the mouse lymphoma forward mutation assay, auranofin at high concentrations (313 to 700 ng/mL) induced increases in the mutation frequencies in the presence of a rat liver microsomal preparation. Auranofin produced no mutation effects in the Ames test (Salmonella), in the in vitro assay (Forward and Reverse Mutation Inducement Assay with Saccharomyces), in the in vitro transformation of BALB/3T3 cell mouse assay or in the Dominant Lethal Assay.

Reproduction Studies

In a fertility study male rats received oral doses of 1, 2 and 4 mg auranofin/kg/day (24/dose) for 63 days prior to mating and through mating. Treatment with auranofin had no effect on mating performance or fertilizing ability.

In a general reproductive performance study female rats received oral doses of 0.25, 0.75 and 3.0 mg auranofin/kg/day (24/dose) for 14 days prior to mating and throughout mating, pregnancy and lactation. Some reduction in body weight gain occurred during pregnancy in the low and high dose groups. A slight reduction in weight gain of the offspring was observed in the middle and high dose groups during the nursing period. There was no adverse effect on estrous cyclicity, sexual receptivity, conception rate, length of pregnancy, general reproductive performance or viability of the offspring.

Auranofin showed teratogenic potential in rabbits. Female rabbits received oral doses of 0.5, 3 and 6 mg/kg/day (31/dose) from days 6-18 of pregnancy. There was a dose-related reduction in food consumption in the middle and high dose groups, and a marked reduction in average live fetal weight in the high dose group. Total occurrences of grossly malformed fetuses were 0.9%, 1.9%, 4.7% and 15.5% for the control, low, middle and high dose groups, respectively. The most common malformations were gastroschisis and umbilical hernia; other malformations observed were limb flexure, exophthalmos, open eyelids, dome-shaped head and edema.

Auranofin had no embryotoxic or teratogenic effect in mice. Female mice received oral doses of 0.2, 1 and 5 mg/kg/day (24/dose) from days 6-15 of pregnancy. Auranofin was not considered to be teratogenic in rats. Female rats received oral doses of 0.5, 2.5 and 5 mg/kg/day (24/dose) from days 6-15 of pregnancy. There was no effect on litter size, birth weight or viability of the offspring.

In a perinatal and postnatal study, female rats received oral doses of 0.5, 2 and 8 mg auranofin/kg/day (24/dose) during the last trimester of pregnancy (beginning on day 15) and throughout lactation. Drug treatment had no adverse effect on late fetal development, the length of pregnancy or delivery of the young. An increase in fetal resorption and decrease in litter size, birth weight, live birth index and viability index were found in the high dose group.

REFERENCES

1. Blechman WJ & Gottlieb NL, (eds.) Proceedings of a Symposium Oral Gold Therapy in Rheumatoid Arthritis: Auranofin, San Francisco, California, Aug 3-4, 1982. In Am J Med December 30, 1983.

2. Blocka K et al. Single dose pharmacokinetics of auranofin in rheumatoid arthritis. J Rheumatol 1982;9(Suppl 8):110-119.

3. Blodgett RC et al. Auranofin: a unique oral chrysotherapeutic agent. Semin Arthritis Rheum 1984;13:255-273.

4. Borg G et al. Auranofin improves outcome in early rheumatoid arthritis. Results from a 2-year, double blind, placebo controlled study. J Rheumatol 1988; 15(12):1747-1754.

5. Capell HA et I, (eds.) Aurfanofin: Proceedings of a Smith Kline & French International Symposium, Amsterdam, November 15-16, 1982. Amsterdam: Excerpta Medica, 1983.

6. Carette S et al. A double-blind placebo-controlled study of auranofin in patients with psoriatic arthritis. Arthritis and Rheumatism 1989;32(2): 58-165.

7. Chaffman M et al. Auranofin: a preliminary review of its pharmacological properties and therapeutic use in rheumatoid arthritis. Drugs 1984;27:378-424.

8. Davis P et al. One-year comparative study of gold sodium thiomalate and auranofin in the treatment of rheumatoid arthritis. J Rheumatol 1985;12:60-67.

9. Gottlieb NL. Comparative pharmacokinetics of parenteral and oral gold compounds. J Rheumatol 1982;9(Suppl 8):99-109.

10. Katz WA et al. Proteinuria in gold-treated rheumatoid arthritis. Ann Intern Med 1984;101:176-179.

11. Morris RW et al. Worldwide clinical experience with auranofin. Clin Rheumatol 1984;3(Supple 1):105-112.

12. Wallin BA et al. Incidence and management of diarrhea during long-term auranofin therapy. J Rheumatol 1988; 15(12):1755-1758.

13. Ward JR et al. Comparison of auranofin, gold sodium thiomalate, and placebo in the treatment of rheumatoid arthritis: A controlled clinical trial. Arthritis Rheum 1983;26:1303-1315.

14. Wenger ME et al. Auranofin versus placebo in the treatment of rheumatoid arthritis. Am J Med I 983; 75(S u ppl 6A):123-127.
